# MEG3 activates necroptosis in human neuron xenografts modeling Alzheimer’s disease

**DOI:** 10.1126/science.abp9556

**Published:** 2023-09-14

**Authors:** Sriram Balusu, Katrien Horré, Nicola Thrupp, Katleen Craessaerts, An Snellinx, Lutgarde Serneels, Dries T’Syen, Iordana Chrysidou, Amaia M. Arranz, Annerieke Sierksma, Joel Simrén, Thomas K. Karikari, Henrik Zetterberg, Wei-Ting Chen, Dietmar Rudolf Thal, Evgenia Salta, Mark Fiers, Bart De Strooper

**Affiliations:** 1 VIB-KU Leuven Center for Brain and Disease Research, 3000 Leuven, Belgium; 2 Leuven Brain Institute, KU Leuven, 3000 Leuven, Belgium; 3 Achucarro Basque Center for Neuroscience, 48940 Leioa, Spain; 4 Ikerbasque Basque Foundation for Science, 48009 Bilbao, Spain; 5 Clinical Neurochemistry Laboratory, Sahlgrenska University Hospital, 431 80 Möndal, Sweden; 6 Department of Psychiatry and Neurochemistry, Sahlgrenska Academy at the University of Gothenburg, 431 80 Möndal, Sweden; 7 Department of Psychiatry, University of Pittsburgh, Pittsburgh, PA 15213, USA; 8 Department of Neurodegenerative Disease, University College London (UCL) Institute of Neurology, London WC1N 3BG, UK; 9 UK Dementia Research Institute at UCL, London WC1E 6BT, UK; 10 Hong Kong Center for Neurodegenerative Diseases, Hong Kong, China; 11 Laboratory for Neuropathology, Department of Imaging and Pathology, Leuven Brain Institute, KU Leuven, 3000 Leuven, Belgium; 12 Department of Pathology, University Hospital Leuven, 3000 Leuven, Belgium; 13 Laboratory of Neurogenesis and Neurodegeneration, Netherlands Institute for Neuroscience, 1105BA Amsterdam, Netherlands

## Abstract

Neuronal cell loss is a defining feature of Alzheimer’s disease (AD), but the underlying mechanisms remain unclear. We xenografted human or mouse neurons into the brain of a mouse model of AD. Only human neurons displayed tangles, Gallyas silver staining, granulovacuolar neurodegeneration (GVD), phosphorylated tau blood biomarkers, and considerable neuronal cell loss. The long noncoding RNA ***MEG3*** was strongly up-regulated in human neurons. This neuron-specific long noncoding RNA is also up-regulated in AD patients. ***MEG3*** expression alone was sufficient to induce necroptosis in human neurons in vitro. Down-regulation of ***MEG3*** and inhibition of necroptosis using pharmacological or genetic manipulation of receptor-interacting protein kinase 1 (RIPK1), RIPK3, or mixed lineage kinase domain-like protein (MLKL) rescued neuronal cell loss in xenografted human neurons. This model suggests potential therapeutic approaches for AD and reveals a human-specific vulnerability to AD.

Mouse models have not allowed us to address the crucial question how the defining hallmarks of Alzheimer’s disease (AD)—amyloid-β (Aβ) plaques, neuronal tau tangles, granulovacuolar neurodegeneration (GVD), and neuronal cell loss—relate to each other. The major problem is that tau pathology in these models can only be induced artificially, by using frontotemporal dementia causing tau mutation or by injecting tau seeds isolated from AD patient brains (1–3). Thus, the fundamental unanswered questions remain as to whether Aβ can induce tau pathology and how neurons die in AD. Unknown human-specific features that escape modeling in rodents are likely underlying this conundrum (4).

Two good models are available that replicate human features of AD: a three-dimensional cell culture based on different types of human cells (5) and xenografted human neurons in mouse brains (6). In this study, we improved the previously Nod-SCID–based xenotransplantation model (6) using the *Rag2^-/-^* (Rag2^tm1.1Cgn^) immunosuppressive genetic background and a single *App^NL-G-F^* (App^tm3.1Tcs^/App^tm3.1Tcs^) knock-in gene to drive Aβ pathology. Human stem cell-derived neuronal progenitor cells (NPCs) transplanted into control *Rag2^-/-^* mice integrated well and developed dendritic spines (fig. S1, A and B). Two months after transplantation, the xenografted neurons already displayed some characteristic mature neuronal (NEUN and MAP2) and cortical markers (CTIP2, SATB2, TBR1, and CUX2). Unlike rodent neurons, human neurons expressed equal amounts of 3R and 4R tau splice forms at 6 months after transplantation (figs. S1C and S5H). Compared to the previously used model (6), the animals showed a considerably longer life span (>18 months longer), allowing for the study of healthy human neurons during brain aging.

We xenografted 100,000 green fluorescent protein (GFP)–labeled H9-derived human cortical NPCs into *Rag2^-/-^/App^NL-G-F^* (hereafter referred to as amyloid mice) or *Rag2^-/-^/App^mm/mm^* (control mice). β sheet staining with dye X34 revealed robust plaque pathology in the amyloid mice (Fig. 1A and fig. S1, D and E). The number of cells (fig. S6H) and transcriptional profile of the human transplants at 2 months were very similar in control and amyloid mice (Fig. 2A). Thus, human neurons integrated and differentiated similarly in control and amyloid mice, in line with the fact that amyloid plaque pathology appeared only after 2 months in this model. Full-blown amyloid plaque pathology was seen at 18 months after transplantation. At this late point in time, human neurons in the control brain appeared generally healthy and displayed neuronal projections and dendritic spines intermingled with host microglial (IBA1) and astroglial (GFAP) cells (fig. S1D). In contrast, grafted neurons in the amyloid mice displayed severe dystrophic neurites associated with microgliosis (six to seven IBA1-positive microglia per Aβ plaque) and astrogliosis (two to three GFAP-positive astrocytes per plaque) (Fig. 1A and fig. S2, A and B). The glial cell recruitment to plaques was very similar to that seen in nongrafted control animals (fig. S3, A and B). Immunohistochemistry with anti–phosphorylated tau (p-tau) antibodies AT8 (p-tau Ser^202^ and Thr^205^), PHF1 (p-tau Ser^396^ and Ser^404^), and MC1 (pathological conformational tau epitope) demonstrated considerable deposition of neuritic plaque tau (NP-tau) (1) in the human neurons of the grafted amyloid mice (Fig. 1, C and D). About 2% (AT8), 5% (PHF1), and 3% (MC1) of the surface was stained with the indicated antibodies in a 20-μm ring around the Aβ plaques (Fig. 1, E to G). Both NP-tau– and AT8-positive neurons appeared as early as 6 months after transplantation (fig. S1E), indicating that amyloid deposition drives tau phosphorylation early on in this model. Notable tau pathology was not observed at 18 months in mouse neurons in the same animal nor in human grafted control or mouse neurons grafted in amyloid mice (Fig. 1, C and D, and figs. S1E, S3, C and H, and S4, A to C).

Pathological, β sheet X34– positive tau reactivity was seen in the xenografted human neurons (fig. S2C). Furthermore, Gallyas silver staining and tau immunogold labeling of PHF fibril-like structures extracted with sarkosyl from grafted neurons in the amyloid mice (Fig. 1, H and I) confirmed the progression of p-tau into pathological states (>20 tau fibrils per electron microscopy grid (control *n* = 4, amyloid *n* = 4) (fig. S6F). Such pathology was largely absent in grafted control or nongrafted amyloid mice (Fig. 1H and figs. S6F and S3, I and J). Finally, and of clinical relevance, we found that the increase in p-tau181 and p-tau231 (7, 8) was statistically significant in the plasma of the grafted amyloid mice but not in control grafted or nongrafted mice (Fig. 1, J and K), reflecting increased secretion of soluble p-tau biomarkers into the bloodstream in response to amyloid pathology, as in human AD.

It is possible to quantify accurately up to 0.1 ng of human DNA in a mixture with 100 ng mouse genomic DNA using quantitative PCR (qPCR) (9) (fig. S6D). Using this assay, we estimated that up to 50% fewer human neurons were present in the amyloid animals compared with controls (Fig. 1L and fig. S6G) at 6 and 18 months after transplantation. Of note, no significant increases in human neurons in the control situation were observed (fig. S6E), and the number of mouse neurons was also not significantly affected in nongrafted control or amyloid mice (fig. S3, E to G). Thus, human, but not mouse, neurons developed AD-like tau pathology upon exposure to amyloid plaques in vivo and degenerated much like their counter-parts in the human brain by an unknown death process.

Previous work has found opposite effects of *Rag2^-/-^* immunodeficiency on amyloid plaque pathology and cellular responses (10, 11). Comparison of soluble Aβ (Aβ_40_ and Aβ_42_) and guanidine-extractable Aβ (Aβ_40_ and Aβ_42_) revealed no significant differences between *Rag2^-/-^/App^NL-G-F^* and *App^NL-G-F^* mice. Bulk RNA sequencing indicated that the main aspects of the innate microglia and astroglial cellular transcriptomic responses to amyloid plaques are maintained in the *Rag2^-/-^/App^NL-G-F^* immunodeficient mouse compared with a previously characterized nonimmunosuppressed amyloid mouse (*APP/PS1*) (12, 13) (Fig. 1B) [coefficient of determination (*R*^2^) = 0.81 for differentially expressed genes]. Thus, although we acknowledge the lack of an adaptive immune system as a weakness of the model, we note that all key features of the neuropathology of AD were reproduced.

## Transcriptional changes in xenografted neurons reveal induction of necroptosis

**Fig. 1:**
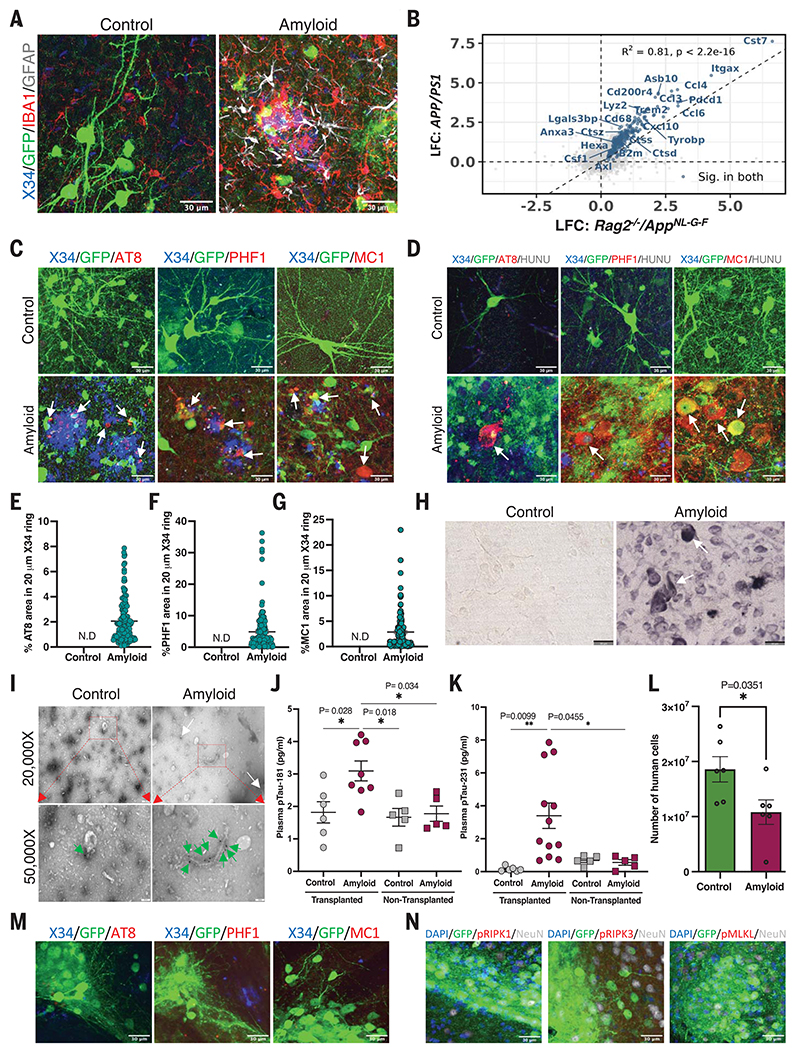
Amyloid plaque deposition is sufficient to induce pathological tau in the grafts. (**A**) Representative confocal images of grafted human neurons of 18-month-old control (*n* = 4) and amyloid (*n* = 4) mice. (**B**) Scatter plot comparing log fold changes (LFC) in *APP/PS1* mice at 10 months and *Rag2^-/-^/App^NL-G-F^* mice at 6 months old. Blue genes are significantly changed in both models (*R*^2^ = 0.81). (**C**) Representative confocal images showing NP-tau and neuronal soma tau (**D**) in 18-month-old control (*n* = 4) and amyloid (*n* = 4) mice. White arrows indicate X34 and NP-tau-positive cells. HUNU, human nucleus. Quantification of (**E**) AT8-positive tau, (**F**) PHF1-positive tau, (**G**) MC1-positive tau around Aβ plaque within 20 μm diameter in 18-month-old animals (*n* = 4, >100 plaques per mice). N.D, not detected. (**H**) Representative light microscope Gallyas silver stain images from 18-month-old control (*n* = 4) and amyloid (*n* = 4) grafted animals. (**I**) Electron micrograph image of immunogold-labeled sarkosyl insoluble tau fibrils isolated from 18-month-old control (*n* = 4) and amyloid (*n* = 4) mice. Tau fibrils are indicated with white (top panel) or green (bottom panel) arrows. (**J**) Quantification of plasma p-tau181 levels (every dot represents one mouse) and (**K**) p-tau231 levels from 18-month-old grafted and nongrafted mice. (**L**) Number of human neurons at 6 months after transplantation in control (*n* = 6) and amyloid (*n* = 6) mice using qPCR. (**M**) Confocal images taken from 6-month-old amyloid animals grafted with stem cell–derived mouse neurons (*n* = 4) did not stain with pathological tau markers (AT8, PHF1, or MC1) or (**N**) mouse-specific necrosome antibodies [pRIPK1(Ser^166^), pRIPK3 (Thr^231^/Thr^232^), or pMLKL (Ser^345^)]. The full panel, along with control mice, is shown in fig. S4, C and D. Scale bars: 30 μm [(A), (C), (D), (M), and (N)], 50 μm (H), 200 nm [(I), top row], 100 nm [(I), bottom row]. Values are presented as mean ± SEM. One-way analysis of variance (ANOVA) with Tukey’s post hoc test for multiple comparisons was used in (J) to (K), and Student’s *t* test was used in (M).

**Fig. 2:**
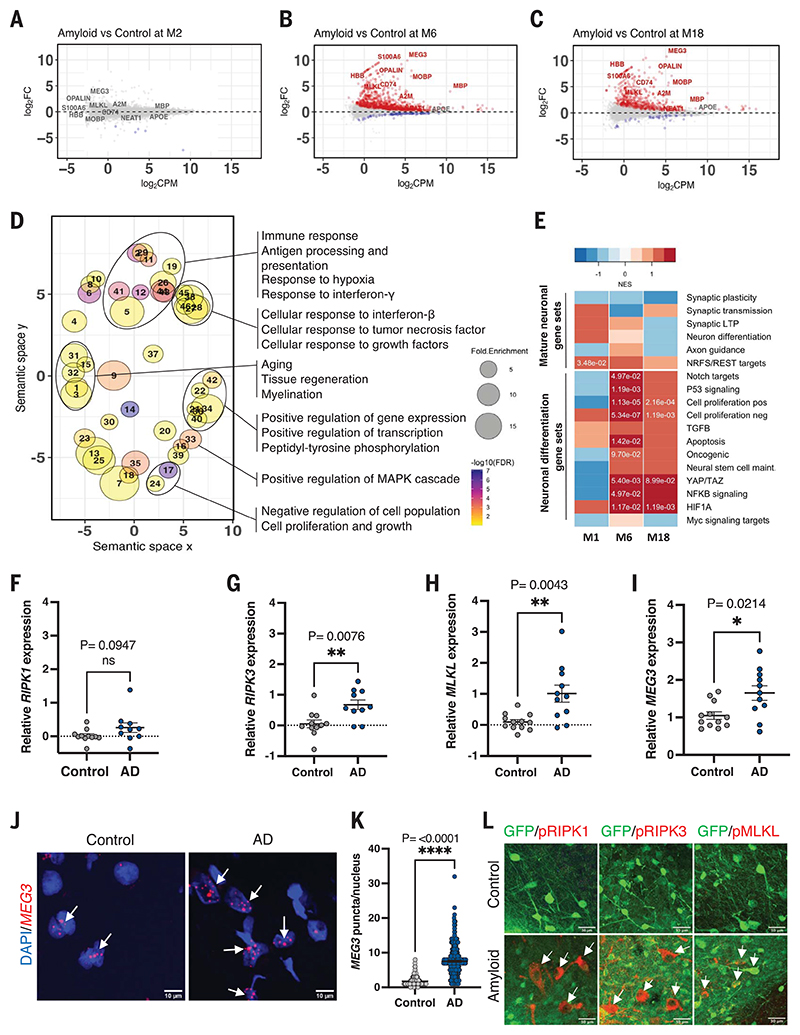
Transcriptional changes in xenografted neurons. Bland-Altman MA plot showing differential expression of genes from RNA sequencing of human grafts from control and amyloid mice at (**A**) 2 months (control *n* = 5, amyloid *n* = 7), (**B**) 6 months (control *n* = 5, amyloid *n* = 5), and (**C**) 18 months (control *n* = 4, amyloid *n* = 3) after transplantation. Red indicates significantly up-regulated genes. Blue indicates significantly down-regulated genes [false discovery rate (FDR) < 0.05]. FC, fold change; CPM, counts per million. (**D**) GO analysis showing terms associated with genes up-regulated at 6 months. Point size represents the fold enrichment of up-regulated genes in the term, and color represents the -log_10_FDR. Only terms with FDR < 0.1 are shown. The *x* and *y* axes represent the “semantic space” (54). (**E**) Heatmap of normalized enrichment scores (NES) in neuronal dedifferentiation gene sets ranked along the differentially expressed genes in the xenografts (amyloid versus control) shown in (A) to (C). Positive enrichments in red, negative enrichments in blue. Significant FDR values (P_adj_ < 0.01) are shown as numbers. (**F** to **H**) Analysis of (F) *RIPK1*, (G) *RIPK3*, and (H) *MLKL* mRNA expression using quantitative reverse transcription polymerase chain reaction (qRT-PCR) on AD (*n* = 11) and control (*n* = 10) postmortem human brain samples. (**I**) Analysis of *MEG3* gene expression using qRT-PCR on AD (*n* = 11) and control (*n* = 10) postmortem human brain samples. (**J**) Confocal images showing *MEG3* RNAscope in the temporal gyrus of AD (*n* = 3) and control (*n* = 2) postmortem human brain samples. Scale bars: 10 μm. (**K**) Number of *MEG3* puncta per nucleus in control (>100 nuclei per sample, *n* = 2) and AD (>100 nuclei per sample, *n* = 3). (**L**) Representative confocal images showing the expression of activated necroptosis pathway markers pRIPK1, pRIPK3, or pMLKL in red (white arrows) in 18-month-old human neurons in control (*n* = 4) and amyloid (*n* = 4) mice. Scale bars: 30 μm. Values are presented as mean ± SEM. Student’s *t* test used in (F) to (I) and (K).

We isolated the transplanted neuron-positive brain regions from mice at 2, 6, and 18 months after transplantation and extracted total RNA for sequencing (mean human reads: ~13.3 million) (fig. S5, A and B). Mapping to mouse and human reference databases generated speciesspecific datasets. As expected, up-regulation of the mouse genes *Axl, B2m, Cst7, Ctss, Itgax, Trem2, Tyrobp, Lyz2, Ccl3*, and *Ccl4* confirmed microglial activation when exposed to amyloid in *Rag2^-/-^/App^NL-G-F^* mice (Fig. 1B; fig. S7, A to C; and table S2) (13). Human samples clustered according to genotype and age (fig. S5C). Differential expression (DE) analysis of the human grafts at 2 months showed only a few significantly down-regulated DE genes of unknown relevance (Fig. 2A and table S1). In contrast, DE of grafts in amyloid and control conditions at 6 and 18 months revealed 916 up-regulated and 73 down-regulated genes, and 533 up-regulated and 34 down-regulated genes, respectively (Fig. 2, B and C, and table S1). Gene alterations of interest included *CD74*, a marker for tau tangle-containing cells (14); *HBB*, a cortical pyramidal neuronal marker (15–17); and *A2M*, which is associated with neuritic plaques in AD human brains (18) (fig. S5E). In addition to the neuronal signature, in line with previous findings (19), we also observed astroglial and oligodendrocytic genes, including *MOBP, MBP, OPALIN, S100A6*, and *APOE*, among others, indicating that the grafted neural stem cells also generated glia in vivo (fig. S5F). A limitation of the current study is the lack of an accurate estimate of the cellular composition of the graft that might influence the transcriptional profile of the grafted cells. Nevertheless, the overall expression profiles of the grafts exposed to amyloid plaques at 6 and 18 months showed strong correlations (*R*^2^ = 0.88), suggesting that the pathological cell states of neurons at 6 and 18 months are similar (fig. S5G). We found a prominent enrichment using GSEA (gene set enrichment analysis) between previously published AD datasets (table S3), including the ROSMAP (Religious Orders Study and Rush Memory and Aging Project) cohort (20), and our data in transplanted neurons at 6 and 18 months after transplantation [adjusted *P* value (*P*_adj_) < 0.05] but not at 2 months (fig. S8, A and B). Furthermore, GSEA also revealed a clear enrichment in up-regulated genes from transplanted neurons and neurons directly reprogrammed from fibroblasts of AD patients (6 months: *P*_adj_ = 1.64 × 10^-5^; 18 months: *P*_adj_ = 2.19 × 10^-4^). These data confirmed that our transplanted neurons capture AD-relevant transcriptional signatures (21).

Functional Gene Ontology (GO) enrichment analysis of significantly up-regulated genes (*P*_adj_ < 0.05) at 6 months using DAVID (Database for Annotation, Visualization and Integrated Discovery) and REVIGO (Reduce and Visualize Gene Ontology) covered semantic space around positive regulation of transcription, protein phosphorylation, positive regulation of mitogen-activated protein kinase cascade, inflammatory responses, including tumor necrosis factor (TNF) and interferon signaling, cell proliferation, aging, tissue regeneration, and myelination (Fig. 2D and table S1). Some reports have suggested that neurons in AD display signatures of hypomaturity, dedifferentiation, and cell cycle reentry (22–24). Using previously published datasets (table S1) and GSEA, we found that cellular signatures of hypomaturity, dedifferentiation, P53, hypoxia-inducible factor α signaling, nuclear factor κB (NF-κB), transforming growth factor-β (TGFβ) signaling, (positive and negative) regulation of cell cycle, and cell cycle reentry were indeed enriched in the 6 and 18 month grafts but not at 2 months (Fig. 2E). Conversely, mature neuronal marker pathways, such as synaptic plasticity, synaptic transmission, long-term potentiation, and axon guidance were not significantly enriched (Fig. 2E).

How neurons die in AD remains controversial (25, 26). As shown in Fig. 1L and fig. S6G, about 50% of xenografted human neurons were lost in the amyloid mice. We did not observe any significant alterations in the expression of genes associated with cell death mechanisms, such as apoptosis or ferroptosis, but found significant up-regulation of *MLKL*, the gene encoding the executor protein of necroptosis (Fig. 2, B and C, and figs. S2E and S6, A to C). This was not observed in the transcriptome of the host tissue (fig. S7, A to C). We used pRIPK1- (Ser^166^), pRIPK3- (Ser^227^), and pMLKL-specific (Ser^358^) antibodies to stain brain tissue from 18-month-old grafted and nongrafted mice. We noticed intense punctuate staining with a vesicular pattern in the soma of the neurons in grafted amyloid mice. These vesicular structures co-stained with casein kinase 1 delta (CK1δ), a marker for granulovacuolar degeneration (Fig. 2L and fig. S6I). Nongrafted control and amyloid animals or mouse neurons derived from NPCs and similarly transplanted into mice did not display these pathologies (figs. S3D and S4D).

We validated this observation by qPCR on RNA extracted from the temporal gyrus of AD and age-matched control brain samples, revealing significant up-regulation of *MLKL* and *RIPK3* (Fig. 2, F to H). We have, on the basis of our initial observations in the xenograft model, extensively confirmed the presence of necroptosis markers in GVD in AD patients in a previous publication (27). Thus, our model has predictive value for the neuropathology of AD.

## *MEG3* modulates the neuronal necroptosis pathway

We identified 36 up-regulated and 21 down-regulated long noncoding RNAs (lncRNAs) in the grafts of 6-month-old animals (table S1). Some of these noncoding RNAs (e.g., *NEAT1*) have been implicated in AD (28), but overall, it is unclear whether or how they contribute to pathogenesis. In our data, the lncRNA *MEG3* (Maternally Expressed 3) was the most strongly (~10-fold) up-regulated gene in human neurons exposed to amyloid pathology (Fig. 2, B to C, and fig. S2D) but not in the host mouse neurons (fig. S7, D to F). *MEG3* has been linked to cell death pathways (29) through p53 (30), is involved in the TGFβ pathway (31), and has been associated with Huntington’s disease (32).

While *MEG3* has not been implicated in AD, we found up-regulation of *MEG3* in AD patient brains in a single-nucleus transcriptomic database (33). We confirmed two- to threefold up-regulation of *MEG3* in RNA extracted from the temporal gyrus of AD patients (table S5) using qPCR (Fig. 2I). *MEG3* in situ hybridization combined with cell-specific immunohistochemical markers NeuN (neurons), IBA1 (microglia), and GFAP (astrocytes) demonstrated its exclusive expression in the nucleus of neurons (fig. S9, A and B) and its strong enrichment in AD brains [2 to 3 puncta per nucleus in control and 8 to 10 puncta per nucleus in AD brain (*P* = <0.0001)] (Fig. 2, J and K). In AD, *MEG3*-expressing neuronal nuclei appeared blotched, with reduced 4′,6-diamidino-2-phenylindole (DAPI) intensity, and *MEG3*-positive neurons also displayed high levels of the necroptosis marker pMLKL (fig. S9C).

**Fig. 3:**
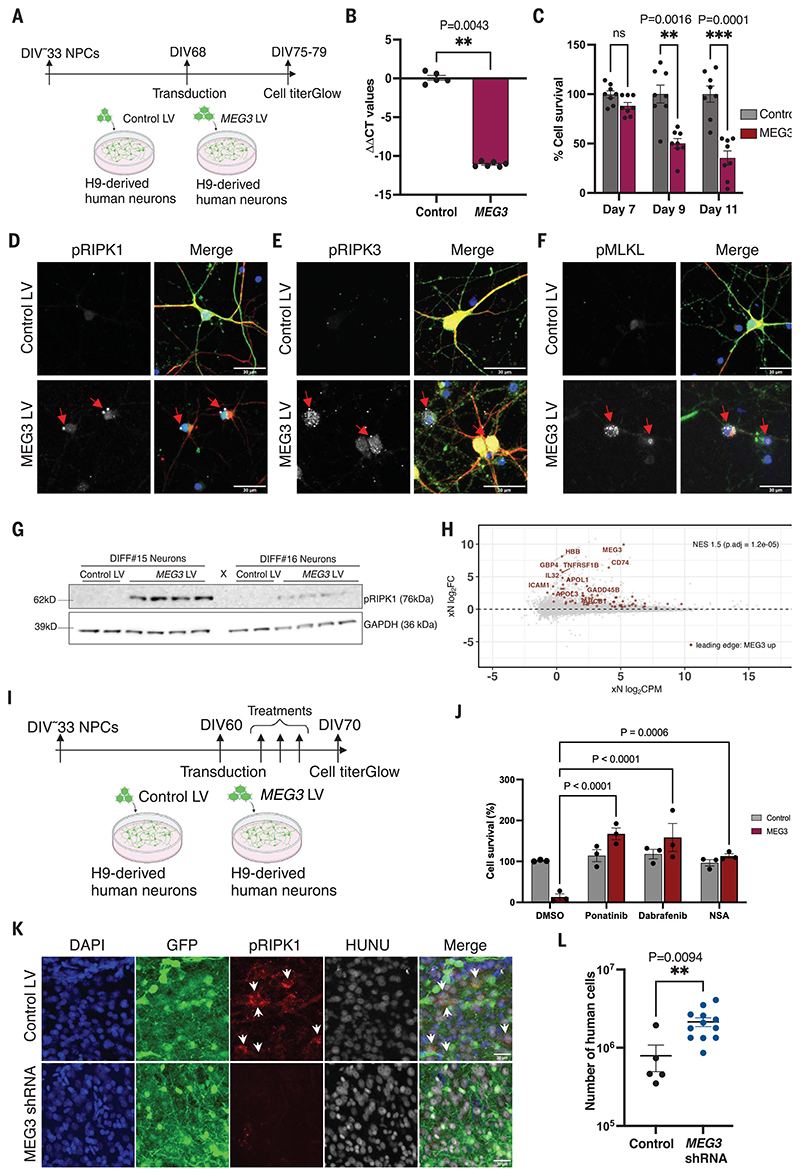
Long noncoding RNA *MEG3* induces necroptosis in human neurons. (**A**) Schematic representation of the *MEG3* expression strategy using lentiviral vectors (LV) in H9-derived human neurons. (**B**) Analysis of the *MEG3* expression 7 days after transduction with control LV (*n* = 5) or *MEG3* LV (*n* = 6) in DIV75 neurons. (**C**) Analysis of neuronal cell survival using Cell-Titer-Glo reagent after transducing with either control LV (*n* = 8) or *MEG3* LV (*n* = 8). (**D**) Confocal images showing pRIPK1 (Ser^166^), (**E**) pRIPK3 (Ser^227^), (**F**) pMLKL (Ser^358^) in neurons at DIV75 and 7 days after transduction with control LV (*n* = 3) or *MEG3* LV (*n* = 3). Scale bars: 30 μm. (**G**) Immunoblot analysis of pRIPK1 (Ser^166^) levels 7 days after transduction (DIV75) with control LV (*n* = 4) or *MEG3* LV (*n* = 8). Glyceraldehyde phosphate dehydrogenase (GAPDH) is the loading control. Two independent differentiations were analyzed. (**H**) Same Bland-Altman MA plot showing differential expression of bulk RNA sequencing of human grafts from 6 months, as in Fig. 2B. Genes highlighted in red are the leading-edge genes identified with GSEA, taking the top 400 up-regulated genes from the bulk sequencing of the primary neurons expressing *MEG3* (fig. S10) and plotting them against the amyloid versus control fold changes of the human grafts (FDR < 0.05). (**I**) Schematic representation of the necroptosis inhibition in vitro using H9-derived human neurons (DIV60) with ponatinib (0.5 μM), dabrafenib (0.9 μM), and NSA (0.5 μM). (**J**) Analysis of neuronal cell survival using CellTiter-Glo reagent after necroptosis inhibitor treatment (*n* = 3). (**K**) Representative confocal images of grafted neurons showing pRIPK1 levels in samples obtained from the 6-month-old animals transplanted with control LV (*n* = 5) or the *MEG3* shRNA (*n* = 5) transduced human NPCs. White arrows indicate pRIPK1-positive cells. Scale bars: 30 μm. (**L**) The human cell number was estimated from 6-month-old xenografted mice from control (*n* = 5) and *MEG3* shRNA (*n* = 12) using qPCR. Values are presented as mean ± SEM. Student’s *t* test used in (B) and (L), one-way ANOVA with Tukey’s post hoc test for multiple comparisons used in (C), and two-way ANOVA with Dunnett’s multiple comparison test used in (J) to measure the statistical significance.

We performed Sanger sequencing on brain cDNA and found that from 15 known transcriptional variants (34), *MEG3* transcript variant 1 (accession number NR_002766.2) was most abundantly expressed in human adult brain. A lentivirus was used to express *MEG3* V1 in H9-derived mature cortical neurons (Fig. 3, A and B), resulting in strong reduction of cell viability at 9 days compared with control (Fig. 3C). Immunohistochemical analysis revealed the presence of activated necroptotic markers pRIPK1 (Ser^166^), pRIPK3 (Ser^227^), and pMLKL (Ser^358^) (Fig. 3, D to F). Necroptosis-positive neurons displayed a reduced amount of cyto-skeletal filament neurofilament-H (NF-H). Immunoblot analysis confirmed increased pRIPK1 (Ser^166^) levels (Fig. 3G). Notably, *MEG3*-induced cell loss was rescued by necroptosis inhibitors ponatinib, dabrafenib, or necrosulfonamide and by CRISPR-mediated deletion of *RIPK1, RIPK3*, and *MLKL* in vitro (Fig. 3, I and J, and fig. S10, H to J).

We performed RNA sequencing of *MEG3*-transduced neurons 7 days after transduction and compared the transcriptomic signatures with those from the xenografted neurons using GSEA. We ranked the genes from the amyloid and control xenografts according to their log fold change differential expression (at 6 months; see Fig. 2B) and compared those to the top up-regulated genes in response to *MEG3* transduction in neurons in vitro. We found a significant enrichment of the up-regulated genes (normalized enrichment score = 1.5, *P*_adj_ = 1.2 × 10^-5^) (Fig. 3H and table S4), suggesting that some transcriptional changes in the xenografted neurons in vivo might be the consequence of up-regulated *MEG3*. DE analysis of *MEG3* transduced neurons revealed no changes in necroptosis genes (fig. S10, A to C). DAVID GO analysis of leading-edge genes from GSEA on up-regulated genes from 6-month transplanted human neurons and in vitro *MEG3*-expressing neurons revealed signatures of NF-κB and TNF signaling, which are related to necroptosis induction (35). In addition, signatures of lipid transport, interferon-γ signaling, positive regulation of peptidyl-tyrosine phosphorylation, and apolipoprotein L signaling were observed (fig. S10D and table S4).

## Inhibition of necroptosis blocks human neuronal loss in the xenografted mice

**Fig. 4:**
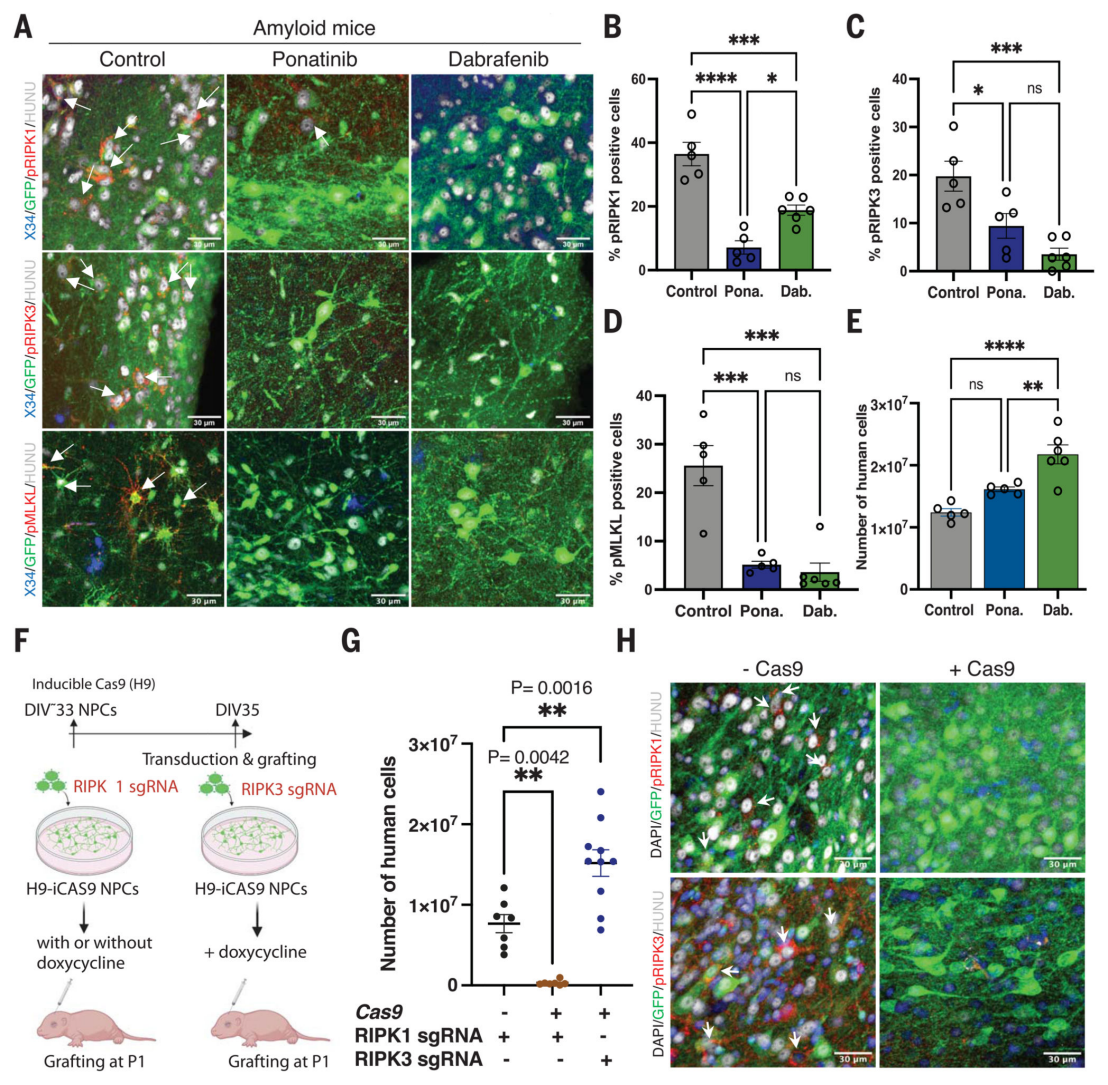
Inhibition of necroptosis prevents human neuronal cell loss in vivo. (**A**) Confocal images showing activated necroptotic markers pRIPKl, pRIPK3, and pMLKL in amyloid animals (*n* = 5) and amyloid animals treated with ponatinib (*n* = 5) or dabrafenib (*n* = 6). White arrows indicate necroptosis marker-positive neurons. Scale bars: 30 μm. (**B**) Quantitative representation of percent of human cells immunoreactive to pRIPKl in control (*n* = 5) and ponatinib-treated (Pona.; *n* = 5) or dabrafenib-treated (Dab.; *n* = 6) animals. Control versus ponatinib: *P* ≤ 0.0001; control versus dabrafenib: *P* = 0.0007; ponatinib versus dabrafenib: *P* = 0.015. (**C**) Percent of human cells showing immunoreactivity to pRIPK3 in control (*n* = 5) and ponatinib-treated (*n* = 5) or dabrafenib-treated (*n* = 6) animals. Control versus ponatinib: *P* = 0.02; control versus dabrafenib: *P* = 0.0007; ponatinib versus dabrafenib: *P* = 0.2. (**D**) Quantitative representation of the percent of human cells showing immunoreactivity toward pMLKL in control (*n* = 5) and ponatinib-treated (*n* = 5) or dabrafenib-treated (*n* = 6) animals. Control versus ponatinib: *P* = 0.0003; control versus dabrafenib: *P* = 0.0001; ponatinib versus dabrafenib: *P* = 0.9. (**E**) Quantitative estimation of the number of human cells in control (*n* = 5) and ponatinib-treated (*n* = 5) or dabrafenib-treated (*n* = 6) grafted mice at 6 months after transplantation. Control versus ponatinib: not significant; control versus dabrafenib: *P* = 0.0001; ponatinib versus dabrafenib: *P* = 0.007. (**F**) Schematic representation of the *RIPK1* and *RIPK3* KO generation and transplantation. (**G**) Number of neurons surviving from 6-month-old mice transplanted with *RIPK1* single guide RNA (sgRNA) without doxycycline (*n* = 7) or *RIPK1* sgRNA with doxycycline (*n* = 7) or *RIPK3* sgRNA with doxycycline (*n* = 10) using qPCR. (**H**) Representative immunofluorescence analysis of phosphorylated necroptosis markers pRIPK1 (*n* = 3) pRIPK3 (*n* = 3) from 6-month-old mice transplanted with *RIPK1* sgRNA or *RIPK3* sgRNA without and with Cas9 induction, as indicated. White arrows indicate pRIPK1- or pRIPK3-positive cells. Scale bars: 30 μm. Values are presented as mean ± SEM. One-way ANOVA with Tukey’s post hoc test for multiple comparisons was used in (B) to (E) and (G).

Blocking *MEG3* up-regulation in NPCs using a previously characterized short hairpin RNA (shRNA) antisense construct (36, 37) significantly improved neuronal survival and was associated with down-regulated expression of necrosome proteins (Fig. 3, K and L, and fig. S10, E to G). It is possible that necroptosis markers are limited to the neurons that are remaining at the stage of pathological analysis. We thus treated xenografted mice (three groups of *n* = 5) from 2 until 6 months after transplantation with the orally available necroptosis kinase inhibitors ponatinib (30 mg/kg) and dabrafenib (50 mg/kg). Ponatinib inhibits RIPK1 (38) and RIPK3 (39) and is a US Food and Drug Administration-approved drug for the treatment of acute lymphoid leukemia and chronic myeloid leukemia. Dabrafenib is a more specific inhibitor of RIPK3 (40). Immunostaining at 6 months revealed a large reduction in levels of pRIPK1, pRIPK3, and pMLKL in both treatment groups compared with untreated mice (Fig. 4, A to D), without alteration of the glial response to amyloid (fig. S9D). qPCR analysis of neuronal cell numbers revealed a significant increase in neurons in the dabrafenib-treated group compared with the control (Fig. 4E). Transplanting *RIPK1* knockout NPCs generated using CRISPR-Cas9, as discussed earlier, demonstrated that neurons were not viable, in agreement with the lethal phenotype of RIPK1 in mouse (41). In contrast, deletion of the *RIPK3* gene significantly improved neuronal survival (Fig. 4, F to H). Immunofluorescence staining confirmed the significant decrease of necrosome signals in *RIPK3*-deficient human neurons.

## Conclusions

Our results demonstrate that amyloid pathology is sufficient to induce hallmark neuropathology of AD, including neuronal tangles and secretion of p-tau181 and p-tau231 biomarkers in blood, by simply exposing human neurons to amyloid plaques. We asked the crucial question of how neurons in AD die. Reports have proposed apoptotic mechanisms, but no convincing evidence has demonstrated that this drives neuronal cell loss in AD (42). Previous publications, one instigated by the observations in the xenograft mice, have confirmed that necroptosis occurs in the AD brain (27, 43). We demonstrate here that neuronal loss can be rescued by treatment with clinically relevant necroptosis inhibitors or by ablating the *RIPK3* gene in transplanted neurons (40). Thus, we suggest that neuronal death in AD is largely driven by necroptosis, linking the loss of neurons to inflammatory processes that are upstream of this well-studied death pathway (13, 44, 45). Therapies that prevent neuronal cell loss, in combination with more-mainstream Aβ- and tau-targeted interventions, might be useful additions to the current efforts to develop disease-modifying strategies for AD (46–48). Necroptosis is an active area of drug development in cancer and ALS (49–51).

Our data suggest that necroptosis is downstream of the accumulation of pathological tau and is induced by the up-regulation of the non-coding RNA *MEG3*, possibly via TNF inflammatory pathway signaling. It needs to be mentioned that the imprinting status of MEG3 and its expression may differ among human embryonic stem cell lines (52). lncRNAs are important regulators of gene expression and influence a variety of biological processes, including brain aging and neurodegenerative disease (53). The large number of noncoding RNAs that are differentially expressed in our AD model warrants further investigation.

It is intriguing that human transplanted neurons display an AD phenotype, whereas mouse neurons interspersed within the graft or neurons derived from mouse NPCs and transplanted in a similar way as their human counterparts do not display such a phenotype. This suggests that unknown human-specific features define the sensitivity of the neurons to amyloid pathology. We noticed that the transplanted human neurons display signatures of down-regulation of mature neuronal properties and up-regulation of immature signaling pathways, which aligns with a recent study analyzing the transcriptional profiles in neurons directly derived from fibroblasts of AD patients (induced neurons) (21). Those neurons did not, however, show tangles, amyloid, or necroptosis markers, and it was therefore not clear how those observations relate to the classical hallmarks of AD. The contribution of these immature signatures to the disease process needs further investigation, but our data indicate that necroptosis is an important contributor to cell death in AD.

Transplanted human neurons, in contrast to transplanted mouse neurons, become diseased when exposed to amyloid pathology. Understanding the molecular basis of the resilience of mouse neurons to amyloid pathology will not only help to model the disease better but might also stimulate research into pathways that protect against neurodegeneration.

## Supplementary Material

Supplementary Materials

## Data Availability

RNA sequencing data are deposited in the Gene Expression Omnibus (accession number GSE195458). *App^NL-G-F^* mice are available from T. Saido under a material transfer agreement (MTA) with RIKEN Institute. PHF1 and MC1 antibodies are available from the P. Davies lab under an MTA with Albert Einstein College. H9 cell lines are available under an MTA with WiCell. All materials used in the current work can be obtained upon request to B.D.S. and will be made available under the standard MTA of VIB.

## Editor’s summary

Neurons are one of the longest-living and enduring cell types of the human body. Balusu *et al*. xenografted human neurons into mouse brains containing amyloid plaques (see the Perspective by Sirkis and Yokoyama). The human neurons, but not the mouse neurons, displayed severe Alzheimer’s pathology, including tangles and necroptosis. Human neurons up-regulated the neuron-specific maternally expressed gene 3 (MEG3) in response to amyloid plaques. Down-regulation of MEG3 protected the neurons from dying in the xenograft model of Alzheimer’s disease. Downstream of MEG3, genetic or pharmacological manipulation of signaling kinases in the necroptosis pathway also protected neurons, suggesting a potential lead toward therapeutic approaches for Alzheimer’s disease. —Stella M. Hurtley

## References

[1] He Z (2018). Nat Med.

[2] Grueninger F (2010). Neurobiol Dis.

[3] Mattsson-Carlgren N (2020). Sci Adv.

[4] Guela C (1998). Nat Med.

[5] Choi SH (2014). Nature.

[6] Espuny-Camacho I (2017). Neuron.

[7] Janelidze S (2020). Nat Med.

[8] Thijssen EH (2020). Nat Med.

[9] Bensalah M (2019). PLOS ONE.

[10] Marsh SE (2016). Proc Natl Acad Sci USA.

[11] Späni C (2015). Acta Neuropathol Commun.

[12] Sierksma A (2020). EMBO Mol Med.

[13] Sala Frigerio C (2019). Cell Rep.

[14] Bryan KJ (2008). Mol Neurodegener.

[15] Biagioli M (2009). Proc Natl Acad Sci USA.

[16] Chuang JY (2012). PLOS ONE.

[17] Brown N (2016). J Mol Neurosci.

[18] Van Gool D, De Strooper B, Van Leuven F, Triau E, Dom R (1993). Neurobiol Aging.

[19] Chen W-T (2020). Cell.

[20] Mostafavi S (2018). Nat Neurosci.

[21] Mertens J (2021). Cell Stem Cell.

[22] Arendt T (2012). Mol Neurobiol.

[23] Arendt T (2000). Ann NY Acad Sci.

[24] Yang Y, Geldmacher DS, Herrup K (2001). J Neurosci.

[25] Wirths O, Zampar S (2020). Int J Mol Sci.

[26] Fünfschilling U (2012). Nature.

[27] Koper MJ (2020). Acta Neuropathol.

[28] Salta E, De Strooper B (2012). Lancet Neurol.

[29] Jiang N, Zhang X, Gu X, Li X, Shang L (2021). Cell Death Discov.

[30] Zhou Y (2007). J Biol Chem.

[31] Mondal T (2015). Nat Commun.

[32] Chanda K (2018). RNA Biol.

[33] Grubman A (2019). Nat Neurosci.

[34] Zhou Y, Zhang X, Klibanski A (2012). J Mol Endocrinol.

[35] Jayaraman A, Htike TT, James R, Picon C, Reynolds R (2021). Acta Neuropathol Commun.

[36] Zhang H (2019). Exp Ther Med.

[37] Bi H (2020). Med Sci Monit.

[38] Fauster A (2015). Cell Death Dis.

[39] Li J-X (2014). Cell Death Dis.

[40] Martens S, Hofmans S, Declercq W, Augustyns K, Vandenabeele P (2020). Trends Pharmacol Sci.

[41] Newton K (2016). Nature.

[42] Roth KA (2001). J Neuropathol Exp Neurol.

[43] Caccamo A (2017). Nat Neurosci.

[44] De Strooper B, Karran E (2016). Cell.

[45] Sierksma A, Escott-Price V, De Strooper B (2020). Science.

[46] Congdon EE, Sigurdsson EM (2018). Nat Rev Neurol.

[47] Sevigny J (2016). Nature.

[48] Mintun MA (2021). N Engl J Med.

[49] Ito Y (2016). Science.

[50] Yuan J, Amin P, Ofengeim D (2019). Nat Rev Neurosci.

[51] Hanson B (2016). Cancer Biol Ther.

[52] Rugg-Gunn PJ, Ferguson-Smith AC, Pedersen RA (2007). Hum Mol Genet.

[53] Lauretti E, Dabrowski K, Pratico D (2021). Ageing Res Rev.

[54] Supek F, Bosnjak M, Skunca N, Smuc T (2011). PLOS ONE.

