# Supplementary material for: MEG3 activates necroptosis in human neuron xenografts modeling Alzheimer’s disease
Source: Science. Author manuscript; Available in PMC 2023 Oct 23. (PMC7615236; doi:10.1126/science.abp9556)
Supplement: Supplementary Materials [file EMS188163-supplement-Supplementary_Materials.zip › science.abp9556_sm.pdf]

Science
AAAS

Supplementary Materials for

MEG3 activates necroptosis in human neuron xenografts modeling Alzheimer's disease

Sriram Balusu *et al.*

Corresponding authors: Sriram Balusu, sriram.balusu@kuleuven.vib.be; Bart De Strooper, b.strooper@ukdri.ucl.ac.uk

Science **381**, 1176 (2023)
DOI: 10.1126/science.abp9556

The PDF file includes:

Materials and Methods
Figs. S1 to S10
References

Other Supplementary Material for this manuscript includes the following:

Tables S1 to S5
MDAR Reproducibility Checklist

Materials and methods

Generation of immunodeficient transgenic amyloid mice

Rag2$^{-/-}$ (*Rag2*$^{tm1.1Cgn}$) and *App*$^{NL-G-F}$ (*App*$^{tm3.1Tcs}$) mice in a C57BL/6 background were housed in our specific pathogen-free (SPF) animal facility. Homozygous *Rag2*$^{-/-}$ single knock-out mice and homozygous *App*$^{NL-G-F}$ transgenic knock-in mice were crossed to obtain the *Rag2*$^{-/-}$/*App*$^{NL-G-F}$ genotype. Isogenic *Rag2*$^{-/-}$/*App*$^{mm/mm}$ (*mm*, Mus *musculus*) containing the *App* wild type allele were used as control mice. Homozygous colonies were maintained. Crosses were conducted within the same genotype to obtain mice for grafting. Mice were randomized, and both sexes were used for the experiments. Mice were housed in groups of four per cage with ad libitum access to food and water and a 14-hour light/10hour dark cycle at 21 °C. For necroptosis inhibition, ponatinib (30 mg/kg) and dabrafenib (50 mg/kg) were mixed in the mouse food, which was replaced every three days during the treatment period. All rodent experiments were approved by the ethics committee of KU Leuven and were executed in compliance with the ethical regulation for animal research.

Pluripotent stem cell culture and neuronal differentiation

Most experiments were performed using the established H9 stem cell line (WAe009-A; https://hpscreg.eu/cellline/WAe009-A), stably expressing GFP under the control of chicken b-actin promoter (CAG promoter). Routine culturing and maintenance of the stem cells was performed on Matrigel coated surface using E8-flex growth media (Thermo; #A2858501). Cells were maintained in a humidified chamber at 37°C with 5% CO_2. Once the cells were confluent, they were passaged using 0.5 mM EDTA. The protocol described by Shi et al., 2012*(55)*, with minor modifications, was used to differentiate stem cells into cortical neurons. On day in vitro (DIV) -2 of neural induction, cells were enzymatically dissociated with StemPro™ Accutase™

Cell Dissociation Reagent (Sigma, # A1110501). Single cells were plated (2 x 10^6/well) in Matrigel-coated 6-well plate in mTeSR1 plus growth medium (STEMCELL Technologies), supplemented with 10 mM Y-27632 ROCK inhibitor (Calbiochem, CA, USA). For neural induction, the culture medium was replaced with a neural maintenance medium (NMM) supplemented with 1 µM StemMACS™ LDN-193189 (Miltenyi Biotec, #130-106-540) and 10 µM SB431542 (STEMCELL Technologies, #72234) every day until DIV12. From DIV12 to DIV24, neuroepithelium was passaged three times using mechanically dissociation, to enrich for neural rosettes. Around DIV27, neural rosettes were dissociated into single cells using the StemPro™ Accutase™ Cell Dissociation Reagent. DIV30 cells were frozen as progenitors (NPCs) for future use.

Human neural progenitor cells (NPCs) culturing for grafting

Grafting experiments of H9-ePSC derived cortical progenitors (NPCs) were performed as described previously (Espuny-Camacho I *et al*., 2017)*(6)*. In brief, time-controlled mating's were performed to obtain pregnant females. Five days before transplantation, frozen neural progenitor cells (NPCs) were thawed in the neural maintenance growth medium and supplemented with ROCK inhibitor (Y-27632; 10 mM Calbiochem, #688000). Cells were incubated in a humidified chamber at 37°C with 5% CO2, with replacement of growth media every 48h until transplantation. On the day of grafting, 30 minutes before enzymatic dissociation, Revitacel (Thermo; #A2644501) was added to the growth medium. Cells were dissociated with StemPro™ Accutase™ Cell Dissociation Reagent (Sigma, # A1110501). The viability of the cells was assessed by trypan blue staining. Finally, the cells were suspended at 100,000 cells/μl^{-1}, or 50,000 cells/μl^{-1} concentration in Leibovitz's L-15 Medium (Thermo; # 11415064) supplemented with 34 mM glucose solution.

Mouse pluripotent stem cell culture, differentiation and grafting

Mouse pluripotent stem cells were differentiated into cortical progenitors following the protocols published in *(56)*. Briefly, mouse ES cells were cultured on 0.1% gelatin in Dulbecco's Modified Eagle Medium (DMEM) supplemented with 15% FBS and Leukemia Inhibitory Factor (LIF, 1000U/ml). To induce differentiation, ES cells were trypsinized and plated on gelatin-coated dishes at a density of 5000 cells/cm2. The day after plating, the culture medium was replaced with DDM media (DMEM/F12+Glutamax #Invitrogen 31331-028; N2 supplement 100x #Invitrogen 17502-048; BSA 7,5% (wt/vol) #Invitrogen 15260-037; 2-mercaptoethanol 50mM #Gibco 31350-010). Cyclopamine (1 mM) was added to the DDM media on day 2 of differentiation. The media with cyclopamine was refreshed every 48 hours until day 10. On day 10, the media was changed to DDM without cyclopamine. on day 15, the cells were dissociated using trypsin and grafted into P1 pups, 100,000 cells mice, similar to human NPC grafting.

Grafting

Grafting was performed as previously described in *(6)*. In brief, grafting took place at the P1-P2 stage. Cryoanesthesia was used for grafting the NPCs. A small incision was made at the injection site (coordinates from bregma: anteroposterior, −1 mm; lateral, ±1 mm) using a sterile surgical blade. For unilateral grafting, 1 µl of the 100,000 cells/µl cell suspension, and for bilateral grafting 1 µl of 50,000 cells/µl was injected using 26G Hamilton syringe, without stereotactic frame support. After the injection, pups were allowed to recover under a heat lamp at 37 °C. Once fully recovered, they were placed back into the cage along with bedding material. Grafted pups were monitored daily for a week.

Sample isolation

For immunohistochemistry of the brain, mice were anesthetized with an overdose of sodium pentobarbital, then perfused transcardially first with PBS followed by 4% PFA in PBS. Collected brains were postfixed overnight in 4% PFA. Brains were embedded in 4% Top Vision low-melting-point agarose (Thermo Fisher Scientific) and cut into 40 µm thick transverse coronal free-floating serial sections using a vibratome (Leica VT1000S). Brain sections were stored in cryoprotectant solution (30% ethylene glycol, 30% glycerol, 40% PBS) at −20 °C until use. For RNA extraction from grafts, animals were euthanized using cervical dislocation. The brain samples were quickly isolated and rinsed in ice-cold PBS containing RNasin (0.2u/ul). Then the brain was sliced into 1 mm thick sections using a cold brain matrix. Sections were collected immediately into cold PBS containing RNasin. These sections were placed under a fluorescent dissection microscope, the fine dissection of the GFP-positive areas was performed, and collected tissue was snap-frozen in liquid nitrogen. The rest of the brain was snap-frozen to be used for detergent insoluble Tau isolations. Samples were stored at -80°C until use. On the day of RNA isolation, samples were thawed on ice, and the Qiagen RNeasy mini kit was used for RNA purification. Blood samples were collected in EDTA-coated tubes during transcardial perfusion. The tubes were incubated on ice for 30 minutes, centrifuged at 2000g, and plasma supernatant was collected. Plasma samples were stored at -80 °C until use.

Immunofluorescence and imaging

For immunofluorescence (IF) analysis, vibratome sections were washed three times with PBS to remove the residual storing solution. Antigen retrieval was performed by microwave boiling in 10 mM tri-sodium citrate buffer pH 6.0 for pRIPK1, pRIPK3 and pMLKL staining's. Then the sections were placed in permeabilization/blocking buffer containing 5% serum, corresponding to the host species of the secondary antibody (Donkey) in PBST (PBS with 0.20% Triton X-100 in

1xPBS) for one hour at room temperature. After the blocking step, the primary antibody (Supplementary Table 5) was added to the blocking solution and gently agitated overnight at 4°C. The next day, sections were washed three times for 5 minutes in PBST. Respective secondary antibodies were added, and sections were incubated for two hours at room temperature. If appropriate, DAPI staining was performed. Subsequently, the samples were washed three times for 5 minutes in PBST and mounted onto glass slides using the Glycergel mounting media and allowed to dry at room temperature. The slides were kept at 4ºC until imaging. Confocal images were obtained using a Nikon Ti-E inverted microscope equipped with an A1R confocal unit driven by NIS (4.30) software. For excitation, 405 nm, 488 nm, 561 nm, 638 nm laser lines were used. All the images were obtained using a 20x (0.75 NA) objective lens, and Z-stack series of images of the area of interest were acquired using the NIS software. Roughly 10-12 Z-sections with 1 mm thickness were obtained. All images were acquired using similar acquisition parameters such as 16-bit, 1024x1024 quality, and images were processed in the FIJI/Image J software. All the images of Z-series stacks were then converted to Fiji/ImageJ maximum intensity projections. For quantification of percent positive cells, three to four sections comprising human xenograft were randomly selected and imaged using the above-described procedure and manually counted using NIS software.

Quantification of the Immunofluorescence images

For quantification of the plaque-associated microglia and astroglia, a 20 µm ring was drawn around the plaque in the Nikon analysis software (NIS-Elements AR, Version 5.21.00). The number of microglia and astrocytes in the 20 µm ring were counted. A semi-automated house-made macro in NIS-Elements AR software was employed for quantifying the NP-Tau in NIS-Elements AR software. In brief, X34-positive plaques were identified with threshold function, and the volume

around the plaque was expanded by 20 µm in the z-stack images. AT8, PHF1, or MC1-positive volume (3D) within the 20 µm area was measured, normalized against the total expanded volume around the plaque, and represented as percent positive volume in the 20 µm X34 ring. N is > 100 plaques per mouse and at least 4 mice from each genotype were used for quantification. For quantifying pRIPK1 (n= 5-6), pRIPK3 (n= 5-6), and pMLKL (n= 5-6) positive neurons, random sections were taken and N>1000 HUNU-positive human neurons and neurons co-labeled with HUNU, and the above-selected markers were counted and represented as percent positive neurons.

Gallyas silver staining

The Gallyas silver staining method was used to stain the Tau tangles*(57)*. The 40 µm vibratome sections were mounted onto positively charged microscope slides (Thermo; #6776214). The sections were allowed to dry for 24 hours before staining. The next day, slides were washed in distilled water for one minute and transferred immediately to alkali silver iodide (4 g sodium hydroxide, 10 g potassium iodide, 3.5 mL of 1% silver nitrate in 100 mL distilled water) for 1 minute. Next, slides were washed 3 times for 1 minute in 0.5% acetic acid. Then the slides were incubated in physical developer solution (Solution 1: 50 mg sodium carbonate in 1 liter; Solution 2: 2 mg ammonium nitrate, 2 mg silver nitrate, 10 mg tungstosilicic acid in one-liter distilled water; Solution 3: 2 mg ammonium nitrate, 2 mg silver nitrate, 10 g tungstosilicic acid, 7.3 ml 35% formaldehyde in one-liter distilled water) for 15-30 minutes. The developer was made fresh before use in a 10:4:6 ratio (solution 1: solution 2: solution 3). Next, the samples were washed in 0.5% acetic acid and 1% sodium thiosulfate for 5 minutes each. Subsequently, samples were washed in distilled water for five minutes, incubated in 0.5% gold chloride solution, and sodium thiosulfate for 5 minutes each. Finally, the samples were washed in distilled water for five minutes,

counterstained with nuclear fast red, and mounted using Permount solution (Fischer Scientific; #15820100).

Insoluble Tau isolation

Sarkosyl insoluble Tau is extracted from the grafted animals as described before*(58)*. Mouse brain samples were homogenized using nine volumes (v/w) of high-salt buffer (10 mM Tris-HCl, pH 7.4, 0.8 M NaCl, 1 mM EDTA, and 2 mM dithiothreitol [DTT], with protease inhibitor cocktail, a phosphatase inhibitor, and PMSF) with 0.1% sarkosyl and 10% sucrose and centrifuged at 10,000 g for 10 min at 4°C. The resulting pellets were reextracted twice using the same buffer, and the supernatants from all three extractions were pooled. Next, sarkosyl was added to the pooled supernatant to reach 1% final concentration. Samples were gently shacken for one hour at room temperature, followed by centrifugation at 300,000 g for 60 min at 4°C. The resulting pellet, which contains 1% sarkosyl-insoluble Tau, was washed once with PBS, and resuspended in PBS (~100µl/gm tissue) by passing through a 27G needle. Then the sample was subjected to a brief sonication (20 pulses at 1 s/pulse) using a water bath sonicator. Samples were centrifuged at 100,000 g for 30 min at 4°C to enrich Tau filaments. The resulting pellet from this step is resuspended in PBS and further sonicated with 60 short pulses (1 s/pulse). Final low-speed centrifugation (10,000 g) was carried for 30 minutes at 4°C to remove contaminating large debris. Finally, enriched Tau PHFs from the supernatant were separated and stored at -80°C until use.

Negative staining and immuno-Electron Microscopy

Immunogold labelling and negative staining of the Sarkosyl insoluble Tau is performed as described before*(59)*. For immuno-EM, diluted sarkosyl-insoluble fractions of the Tau filaments were loaded onto the glow-discharged (Leica EM AC600) 400 mesh formvar/carbon film-coated

copper grids (#01754-F; Ted Pella Inc) for 5 minutes and washed three times with PBS and blocked for 10 min with 5% acetylated BSA (Aurion). The grids were incubated with primary antibodies in a blocking buffer for three hours at room temperature followed by three times five minutes wash with PBS. Then the grids were incubated with 10 nm gold-conjugated secondary antibodies (1:20) in blocking buffer for 2-3 hours at room temperature. After that, grids were washed three times with sterile water and stained with 2% uranyl acetate for one minute. Finally, the grids were dried and imaged using a JEM-1400 transmission electron microscope (JEOL), equipped with an 11Mpixel Olympus SIS Quemesa camera.

Cell number determination using qPCR

The number of human cells in the xenografted mice was determined as described previously*(9, 60, 61)*. In brief, total DNA from the xenografted mice was extracted using the TRI Reagent™ Solution (Thermo Fischer, #AM9738) according to the manufacturer's instructions. Using human-specific primers, a standard curve was established with human genomic DNA (gDNA) alone. The specificity of the primers was assessed by spiking an increasing amount of mouse gDNA. 80ng of DNA from the xenografted mice samples was used for the final qPCR assay. An absolute number of human cells was calculated by interpolating the standard curve and considering the standard reference of 6 pg of DNA/cell in the diploid human cell.

RNA extraction from human brain

For qPCR analysis, frozen brain samples from the superior occipital gyrus or superior parietal gyrus of AD and age-matched control samples obtained from Netherlands Brain Bank were used (Supplementary Table 5). According to the manufacturer's instructions, RNA was extracted from the brain samples using TRI Reagent™ Solution (Thermo Fischer, #AM9738).

RNAScope

Cryostat sections from fresh frozen brain samples from the temporal gyrus (Supplementary Table 5) were used for the RNAScope based detection. RNAScope was performed according to the manufacturer's instructions using a human *MEG3* -specific probe (RNAscope™ Probe- Hs-MEG3-No-XMm Cat No. 523401).

Plasma p-Tau181 or p-Tau231 measurements

Plasma samples were thawed at room temperature, vortexed for 30 seconds, and then centrifuged for 10 minutes at 4000 *g* at room temperature. Plasma p-Tau181 and p-Tau231 concentration was measured at the Clinical Neurochemistry Laboratory, University of Gothenburg (Mölndal, Sweden) in one analytical run on a Single-molecule array (Simoa) HD-X instrument (Quanterix, Billerica, MA, USA). All experimental samples were run in singlicate after diluting the samples 1:2 in Tau 2.0 buffer (Quanterix, Billerica, MA). At the beginning and end of the assay plate, two internal quality control samples with concentrations of 11.1 pg/mL and 4.0 pg/mL were run in duplicate, with intra-plate variability of 2.2% and 1.5 %, respectively. AT270 or AT180 mouse monoclonal antibody (Invitrogen, Waltham, MA, USA) specific for the threonine-181 or threonine-231 phosphorylation site was coupled to paramagnetic beads (Quanterix) and used for capture. As a detector, the anti-Tau mouse monoclonal antibody Tau12 (BioLegend, San Diego, CA, USA) was used, which binds the N-terminal on human Tau protein. The detection antibody was conjugated to biotin (Thermo Fisher Scientific, Waltham, MA, USA) following the manufacturer's recommendations. Full-length recombinant Tau 1-441 phosphorylated *in vitro* by glycogen synthase kinase 3β (GSK3β) (SignalChem, Vancouver, BC, Canada) was used as the calibrator.

Protein concentration determination

The total protein concentration of the protein samples was determined with the standard Bradford protein assay.

Immunoblotting

25-30 μg of protein was used for immunoblotting. Samples were diluted with sterile water up to 12 μl, and 4 μl of NuPAGE™ LDS Sample Buffer (4x) (Thermo Fisher Scientific), supplemented with 4% beta-mercaptoethanol. Samples were boiled for 10 min at 70°C. Denatured samples were loaded onto a 4-12 % Bis-Tris Gel (Invitrogen) and run at 170 V for 1 h in NuPAGE™ MOPS SDS Running Buffer (Thermo Fisher Scientific). Proteins were transferred to a 0.2 μm nitrocellulose blotting membrane (GE Healthcare) for one hour at 25 V in cold transfer buffer (NuPAGE™, Thermo Fisher Scientific) with 10 % MeOH. Ponceau staining was performed, and the membranes were blocked for one hour with 5 % milk in 0.1 % Tris buffered saline (TBS) Tween20 (TBS-T). The primary antibodies were added in blocking solution and incubated overnight at 4°C. The next day, the membranes were washed three times with TBS-T and incubated for one hour at room temperature with the appropriate secondary antibodies (Supplementary Table 5). Blots were developed by adding Western Lightning Plus-ECL Substrates (PerkinElmer) and imaged with an ImageQuant LAS4000 (GE Healthcare). For semi-quantitative analysis of western blots, AIDA Image Analyzer v5.0 was used.

Neuronal cultures and inhibitor treatment

H9-derived NPCs (~DIV30) were thawed on PLO-Laminin coated plates. After NPCs were recovered, they were treated with 10 μM DAPT (Sigma-Aldrich, #D5942) for two days to facilitate

faster maturation. After DAPT treatment, NPCs were maturated in neuronal maintenance media (NMM) for 15 days. For the necroptosis inhibitor experiments neurons were treated with Ponatinib (0.5 μM), Dabrafenib (0.9 μM) or necrosulfonamide (NSA, 0.5 μM), starting 3 days post transduction with either control or MEG3 lentiviral vectors. Treatment continued every 48 hours with fresh media changes until the cell death assay was performed.

MEG3 expression

*MEG3*expression was achieved by cloning the cDNA of MEG3 transcript variant-1 under EF1α promoter in a lentiviral vector backbone (VectorBuilder). A lentiviral vector containing CAG: GFP was used as a control vector. For expression in neurons, NPCs were thawed on PLO (Sigma-Aldrich, # P4957) and laminin (Sigma-Aldrich, #L2020) coated plates. NPCs were treated with 10 μM DAPT (Sigma-Aldrich, #D5942) for two days to induce neurogenesis. Neurons were transduced with either control or *MEG3* construct at ˜DIV68.

MEG3 knockdown

Human *MEG3* knockdown was achieved using shRNA. The MEG3 shRNA sequence was obtained from published literature *(36, 37)*. MEG3 shRNA sequence 5′-GAGAGGTTGTTTCACTGGTATCTATTGCA-3′ was cloned into the Lentiviral mammalian shRNA knockdown vector under U6 promoter. Additionally, EGFP together with puromycin selection markers were also cloned under the hPGK promoter. Neuronal progenitors (NPCs) derived from H9 cells were transduced with either the control or the MEG3 shRNA lentiviral vectors. Following transduction, cells were selected using puromycin selection marker before grafting. 100,000 cells/mice were grafted on P1, similar to H9 NPCs.

CRISPR mediated RIPK1, RIPK3 or MLKL deletion

Doxycycline Inducible Cas9-GFP stem cells were transduced with sgRNAs against RIPK1 (CCATGCGGCTGCCATAAAGA), RIPK3 (GTTTGTTAACGTAAACCGGA), or MLKL (CCTGTTTCACCCATAAGCCA) cloned into the lenti-Guide-Puro (Addgene #52963). After transduction stem cells were selected with the puromycin to eliminate non-transduced cells. After selection, cells were treated with doxycycline (2 μg/ml) for 3 days to induce the Cas9. T7-endonuclease assays were performed to ensure the INDEL formation at the respective sgRNA target location. Once the Cas9 INDEL activity was confirmed, the stem cell clones were differentiated into neural progenitor cells (NPCs) as previously described. Before transplantation, the NPCs were treated with doxycycline (2 μg/ml) for 3-4 days to induce Cas9 expression. The treated NPCs were then transplanted into neonatal subjects at postnatal day 1 (P1), following the protocol mentioned above. Only sgRNAs that demonstrated efficacy in downregulating the target protein or inducing INDEL formation at the sgRNA binding region were selected for the experiments.

Cell viability

Cell viability experiments were performed using the CellTiter-Glo® Luminescent cell viability assay, according to the manufacturer's instructions. In brief, cells were seeded in a 96-well plate and transduced with either control or *MEG3* vector at MOI-10. On the day of the assay, conditioned media was removed, and fresh 100 μl media was added along with 100 μl of CellTiter-Glo® reagent. Cells were incubated at room temperature for 30 minutes on a shaker before reading the luminescence signal on an Envison plate reader. The data of the control-DMSO group or control virus-infected group is set at 100%, then every other well within the same plate is normalized to this 100% survival.

RNA-Seq alignment and pre-processing

For xenografted samples, reads were aligned to a joint human plus mouse reference genome (GRCh38 and mm10, build 102) using STAR v2.7.3*(62)*, and then counted using Subread's featureCounts v2.0.1*(63)*, in both cases using default parameters. Read count matrices for human and mouse were treated separately for downstream analysis. We filtered out genes with a mean read count < 5. We achieved means of 13,355,157 human reads per sample, and 162,882,734 mouse reads per sample. For in vitro *MEG3* overexpressing samples, reads were aligned to the human GRCh38 reference (build 102) and counted as above. After filtering low-count genes (as for xenografted samples), a mean read count of 20,770,413 reads per sample remained (Fig. S5). Raw counts were normalized using the R package edgeR v3.34, specifically a weighted trimmed mean of M values (TMM)*(64)*. Multidimensional scaling (MDS) was performed on the normalized count data using the plotMDS() function in edgeR. Xenograft sample (SB295) appeared as an outlier in both mouse and human and was excluded from the analysis (fig. S5C-D). In the human data, SB197 and SB198 also separated out from other 2M samples. These two samples showed strong upregulation of cell cycle markers relative to the other 2M samples. They were not excluded.

Differential expression

Differential expression (DE) analysis was performed using edgeR's generalized linear model, testing for differential expression using likelihood ratio tests. False Discovery Rate < 0.05 was considered significant. For *MEG3* expression data, we set differentiation run as a covariate, as it was shown to be a confounder in an MDS analysis (fig. S10B). DE results are available in Supplementary table 4. For visualizing the data, Bland–Altman plot (MA) were used.

Determination of Tau Splice Form Usage

Tau isoform ratios were calculated as described in *(6)*. Raw junction counts generated by the STAR aligner *(62)* were collected and normalized for library size using Limma Voom *(65)*. We extracted the following normalized junction counts from MAPT using the GRCh38 reference: exon 9-10 (X9,10, chr17, position 45,996,665 to 46010309), 9-11 (X9,11, chr17, position 45,996,665 to 46,014,242) and 10-11 (X10,11, chr17, position 46,010,403 to 46,014,242). The 3R splice isoform is represented by the junction 9-11, and 4R by both 9-10 and 10-11. The percentage of MAPT 4R usage was subsequently calculated by: 100 * (X9,11) / (0.5 * (X9,10 + X10,11)).

Comparison of $Rag2^{-/-}$ and* non-*$Rag2^{-/-}$ mouse transcriptomes

We compared the transcriptomic responses in the *$Rag2^{-/-}/App^{NL-G-F}$* response to a previously documented amyloid mouse model response*(12)*. We extracted differential expression results from $APP^{swe}/PS1^{L66p}$ versus controls at 10 months. We plotted these log fold changes against Rag*$2^{-/-}/App^{NL-G-F}$* log fold changes at 6M, using only genes present in both datasets. A correlation was calculated between genes that were significantly differentially expressed in one or both studies.

Functional Enrichment Analysis

DAVID (The Database for Annotation, Visualisation and Integrated Discovery) was used to perform functional enrichment*(66, 67)*. REVIGO*(54)* was used for visualizing gene ontology terms in semantic space, setting the ‘species’ option to *homo sapiens*, and using their SimRel algorithm to cluster similar terms. Terms with FDR < 0.1 were considered significant unless otherwise indicated. Functional enrichment results are available in Supplementary Table 1 for transplanted neurons. Functional enrichment results for *MEG3* upregulated and downregulated genes are in Supplementary Table 4 for transplanted neurons.

Gene set Enrichment Analyses

GSEA was performed using the fgsea package in R (v1.18.0) with default parameters, using the Benjamini-Hochberg correction. When searching for enrichment in xenografted neurons, we used the log fold changes from human xenografted neurons in amyloid mouse versus their controls, at each time point, as ranks. Genesets used in these analyses are described below. When searching for enrichment in in *vitro MEG3*-transduced cortical neurons, we used the log fold changes of *MEG3*-expression versus controls.

Gene sets used

For the dedifferentiation analysis, genesets were taken from*(20, 22–24, 68-69)*. Genesets are available in Supplementary Table 3. Genesets related to comparison of transplanted neurons with bulk AD brain transcriptome, related to Extended data fig. 8a, b are provided in the Supplementary Table 3. For the gene set enrichment analysis of MEG3 transduction in xenografted neurons, we selected the top 400 *MEG3*-expression upregulated genes, based on log fold change, as our gene sets.

Supplementary Figure 1:

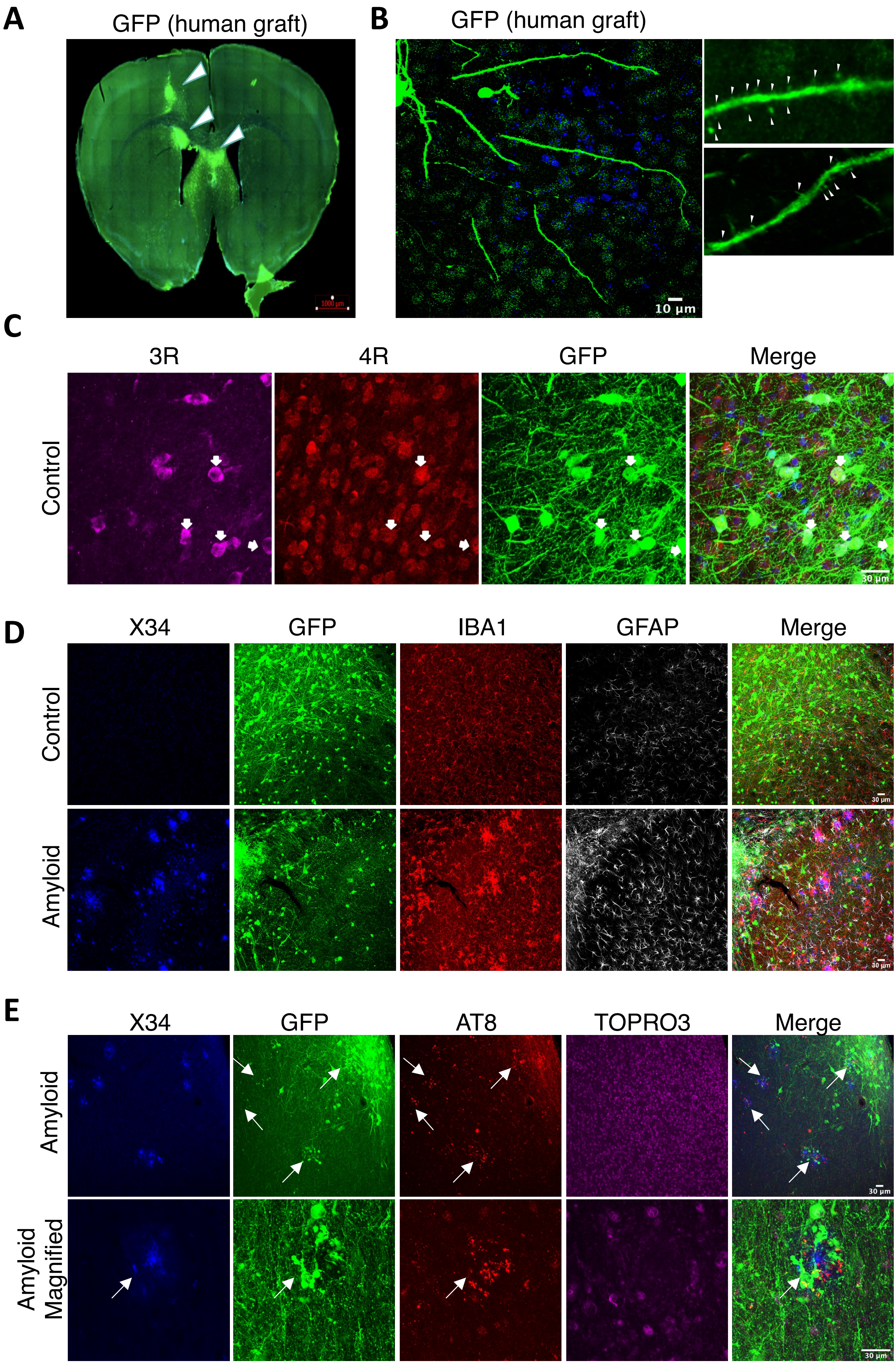

Supplementary Figure 1: Integration of the transplanted neurons in the Rag2$^{-/-}$ animals.

(A) Coronal section of 18-months old grafted mouse brain showing the graft location (green, indicated with white arrows). Scale bar 1000 µm. **(B)** Higher magnification image of neuronal process displaying dendrites and mature spines (right boxes). White arrows indicates dendritic spines. Scale bar 10 µm. **(C)** Representative confocal images of grafted neurons 6 months post-transplantation stained with 3R (magenta) and 4R (red) Tau isoforms (examples of human neurons indicated with white arrows). Scale bar 30 µm. **(D)** Representative confocal images showing glial responses at 18 months post-transplantation. Amyloid (X34 in blue), human neurons in green (GFP), GFAP (grey), IBA1 (red). Scale bar 30 µm. **(E)** Representative confocal images 6-months old grafted mice showing dystrophic neurites and neuritic plaque Tau (NP-Tau) pathology (AT8) around the X34 plaques 6 months post-transplantation. The top panel shows NP-Tau, indicated with white arrows. The bottom panel shows a magnified region of one plaque showing dystrophic neurites and NP-Tau. Amyloid (X34 in blue), human neurons in green (GFP), P-Tau (AT8, red), TOPRO3 (nucleus, magenta). Scale bar 30 µm.

Supplementary Figure 2:

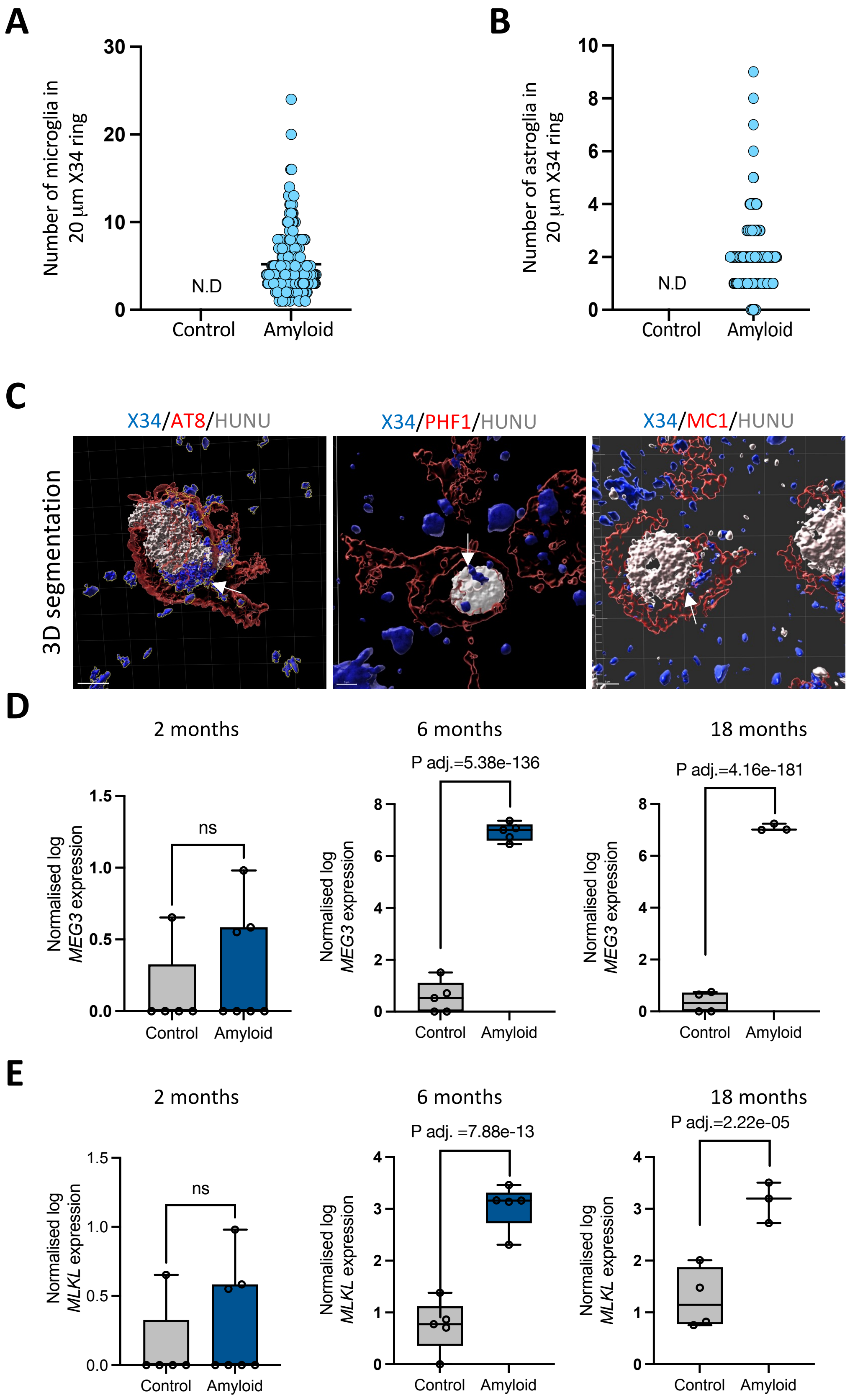

Supplementary Figure 2: Characterization of the 18 months old grafted animals.

(A) Quantification of the number of host microglia around the Aβ plaque, within 20 microns diameter (n=4, >100 plaques/mice). **(B)** Quantification of the number of host astrocytes around the Aβ plaque, within 20 microns diameter (n=4, >100 plaques/mice). **(C)** Neurons immunoreactive to AT8, PHF1 or MC1 are segmented in Imaris software and rendered to reveal intracellular X34 staining (β-sheet fibrillary structures), an indicative of the intracellular Tau fibrils. Extracellular X34 (blue) staining represents Aβ plaques where as intracellular X34 (blue) represents Tau β-sheet structures. White arrows indicate X34 staining in neuronal somas. Amyloid (X34, blue), P-Tau (red), human nucleus (HUNU, grey). Scale bar 5 µm. **(D)** Human *MEG3* expression from 2-months (control n=5, amyloid n=7)**,** 6-months (control n=5, amyloid n=5), and 18-months (control n=4, amyloid n=3) post-transplantation. **(E)** Human *MLKL* expression from 2-months (control n=5, amyloid n=7)**,** 6-months (control n=5, amyloid n=5), and 18-months (control n=4, amyloid n=3) post-transplantation.

Supplementary Figure 3:

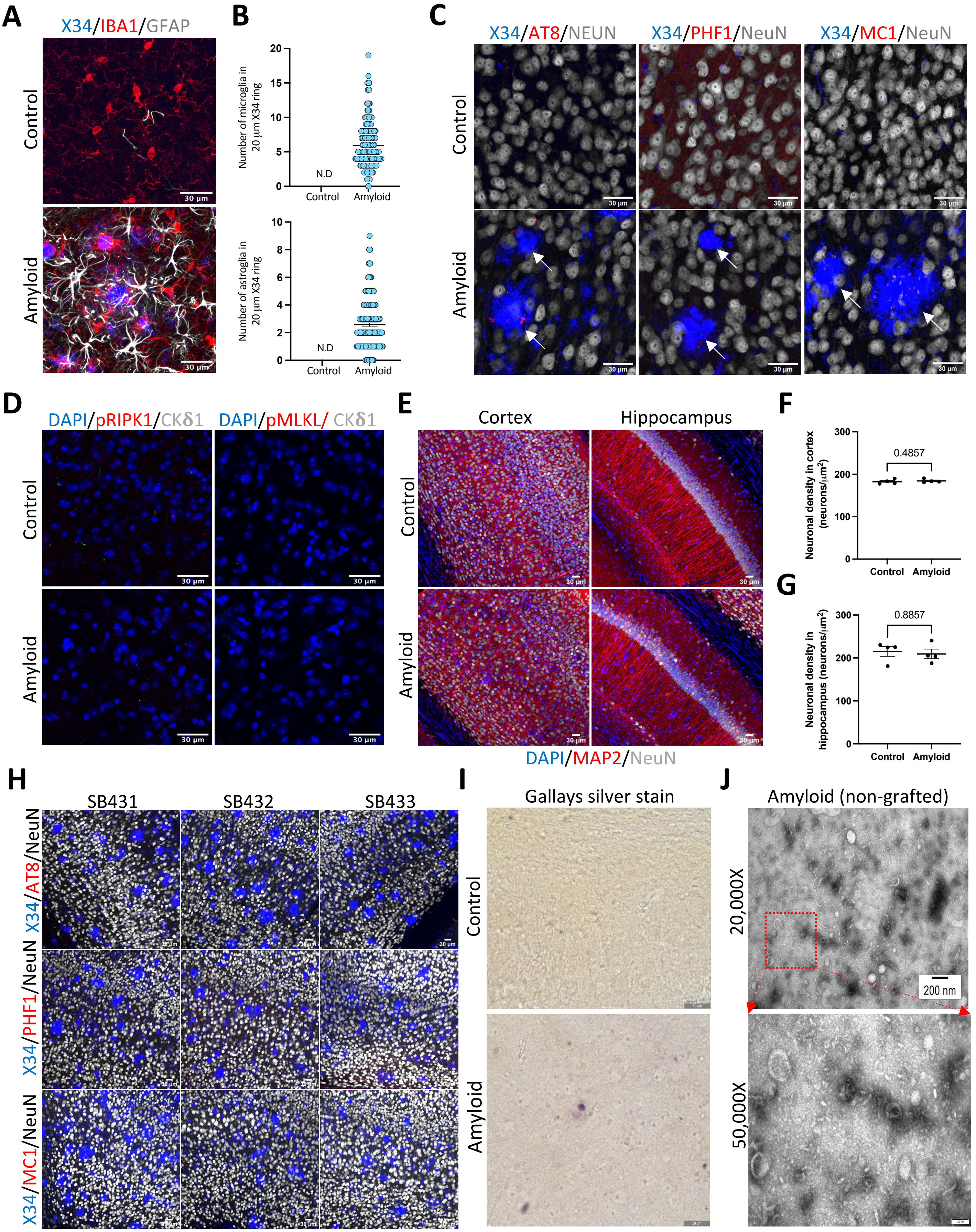

Supplementary Figure 3: Characterization of 18 months old non-grafted animals.
(A) Representative confocal images of non-grafted 18 months old control (top) (n=4) and amyloid mice (bottom) (n=4) showing the glial reaction. Amyloid (X34, blue), microglia (IBA1, red), Astrocytes (GFAP, grey). Scale bar 30 µm. **(B)** Quantification of the number of host microglia (top) and astroglia (bottom) around the Aβ plaque, within 20 microns diameter (n=4 mice, >100 plaques/mice). ND, not observed. **(C)** Representative confocal images of Tau showing the absence of AT8 (P-Tau Ser202, Thr205), PHF1 (P-Tau Ser396), and MC1 (pathological conformation) in 18 months old control (n=4) and amyloid (n=4) animals. Amyloid (X34, blue), P-Tau (AT8 or PHF1, red), pathological conformation of Tau (MC1, red), neurons (NeuN, grey). Arrows indicate plaques. Scale bar 30 µm. **(D)** Representative confocal images showing absence of activated necroptotic markers from 18-months old control (n=4) and amyloid (n=4) mice. Amyloid (X34 in blue), pRIPK1 and pMLKL (red), GVDs (CK1**δ**, grey). Scale bar 30 µm. **(E)** Representative confocal images displaying neuronal density from cortex and hippocampus of the 18-months old control (n=4) and amyloid mice (n=4). Nucleus (DAPI, blue), MAP2 (red), neurons (NeuN, grey). Scale bar 30 µm. **(F)** Quantitative representation of the neuronal density in cortex in 18-months old control (n=4) and amyloid (n=4) mice. Mann-Whitney test. **(G)** Quantitative representation of the neuronal density in hippocampus in 18-months old control (n=4) and amyloid (n=4) mice. Mann-Whitney test. **(H)** Representative confocal images of large field of cortex from 18 months old non-transplanted amyloid mice showing absence of AT8, PHF1 or MC1 staining (n=3). Amyloid stain X34 in blue, AT8, PHF1 or MC1 in red, NeuN in white. Scale bar 30 µm. **(I)** Represenative light microscopic images of Gallays silver stain from 18-months old non-grafted control and amyloid animals. Scale bar 50 µm. **(J)** Representative immuno electron micrograph images of immunogold labelled sarkosyl insoluble fractions from 18-months old amyloid mice (n=4). Top panel indicates 20,000x magnification (scale bar, 200 nm), bottom panel indicates 50,000x magnification (scale bar, 100 nm).

Supplementary Figure 4:

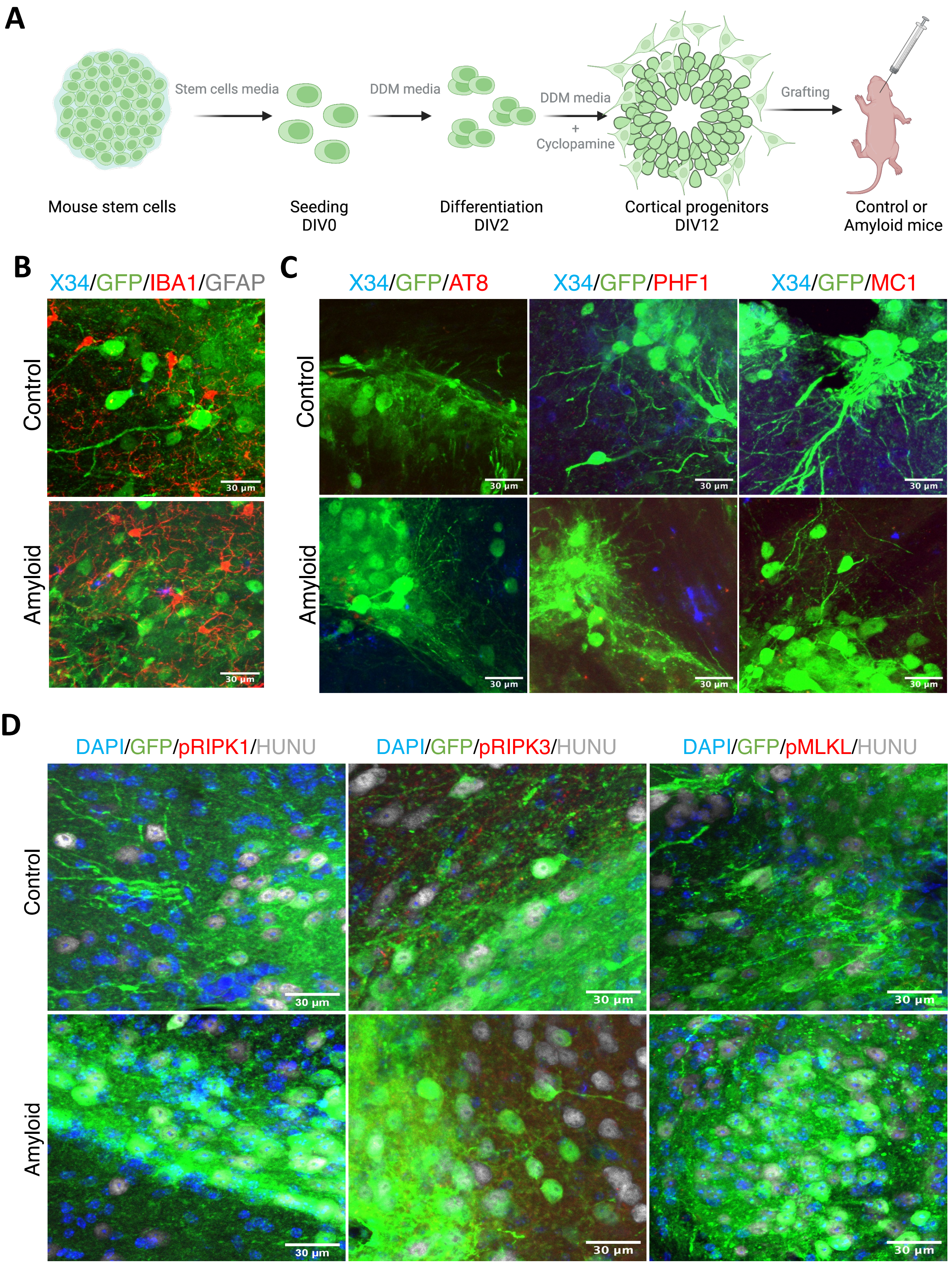

Supplementary Figure 4: Characterization of Tau pathology and necroptosis signatures in 6-months old animals grafted with mouse NPCs.

(A) Schematic representation of the mouse neural progenitors' cells differentiated from the mouse embryonic stem cell line (E14 cell line, GFP positive) following the protocols published in Gaspard et al 2009. **(B)** Representative confocal images of grafted mouse neurons from 6-months old control (top, n=4) and amyloid (bottom, n=4) mice. Amyloid (X34, blue), grafted mouse neurons (GFP, green), microglia (IBA1, red), astrocytes (GFAP, grey). Scale bar 30 µm. **(C)** Representative confocal images taken from 6-months old mouse neuron grafted control (n=4) and amyloid (n=4) animals stained with either AT8, PHF1 or MC1. Amyloid (X34, blue), grafted mouse neurons (GFP, green), AT8 (P-Tau Ser202-Thr205, red), PHF1 (P-Tau Ser396 and Ser404, red), MC1 (pathological conformation, red) and neurons (NeuN, grey). Scale bar 30 µm. **(D)** Representative confocal images taken from 6-months old mouse neuron grafted control (n=4) and amyloid (n=4) animals stained with mouse specific necrosome antibodies. Grafted mouse neurons (GFP, green), pRIPK1(S166), pRIPK3 (T231/232) or pMLKL (S345) in red. Scalebar 30 µm.

Supplementary Figure 5:

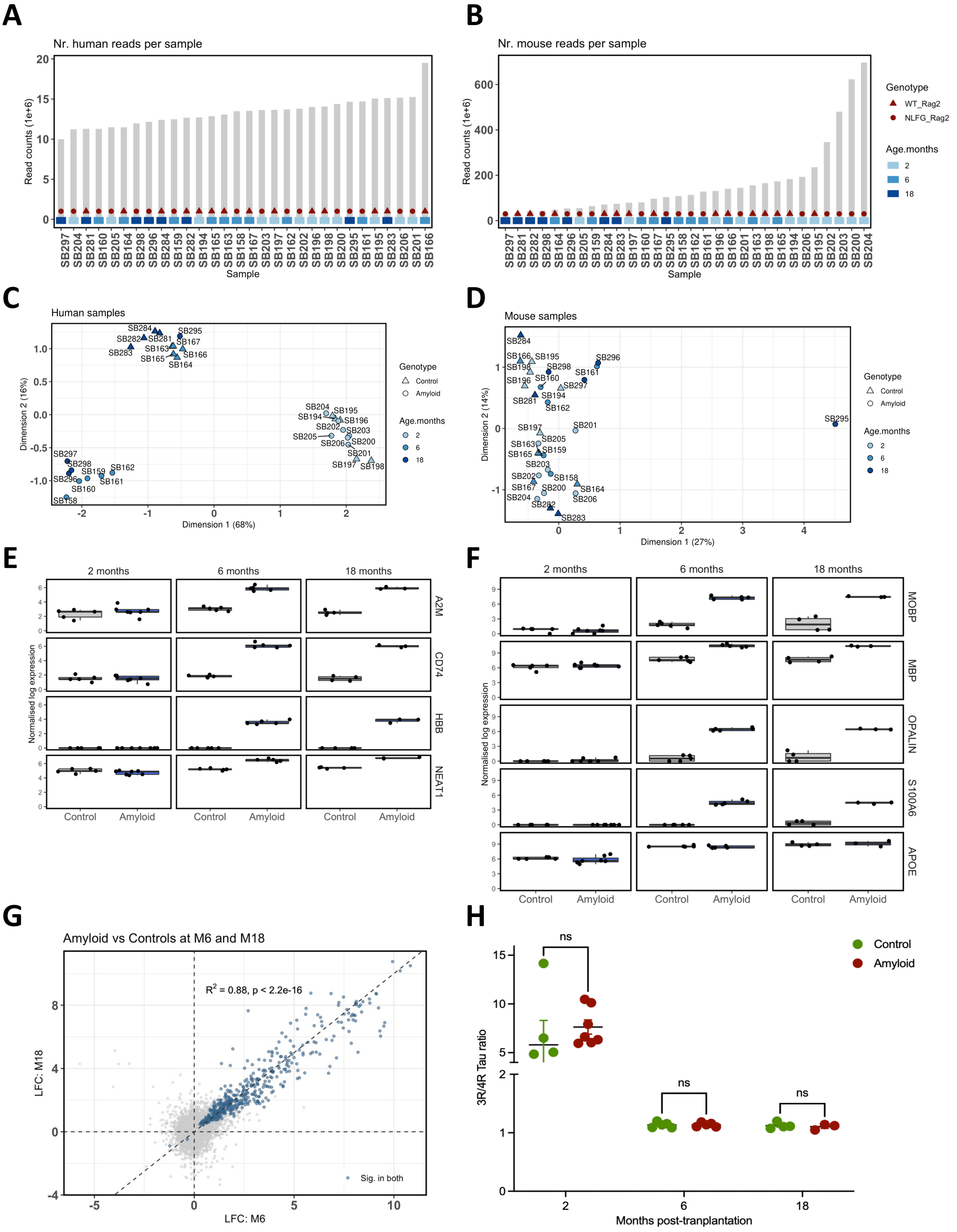

Supplementary Figure 5: RNA sequencing of the human graft 2-, 6– and 18-months post-transplantation. **(A)** Representation of the total number of reads obtained from human graft in control and amyloid animals 2-months (control n=5, amyloid n=7)**,** 6-months (control n=5, amyloid n=5), and 18-months (control n=4, amyloid n=4) post-transplantation. **(B)** Representation of the total number of reads obtained from the host (mouse) tissue from 2-months (control n=5, amyloid n=7)**,** 6-months (control n=5, amyloid n=5), and 18-months old (control n=4, amyloid n=3) animals. **(C)** Multi-dimensional scaling (MDS) of the bulk RNA data from human grafts. **(D)** Multi-dimensional scaling (MDS) analysis of the bulk RNA data from host mouse tissue. **(E)** Normalized gene expression levels of *A2M, CD74, HBB, NEAT1*, and *MOBP* in transplanted neurons at 2-, 6-, and 18-months post-transplantation. **(F)** Log normalized gene expression levels of MOBP, *MBP, OPALIN, S100A6,* and *APOE* in transplanted neurons at 2-, 6-, and 18- months post-transplantation. **(G)** Scatter plot showing comparison of log2 fold changes (LFC) in human neurons between 6M and 18M. Genes highlighted blue are significant in both timepoints. The Pearson correlation (R^2) of genes significantly changing in both models is 0.88. **(H)** Analysis of human MAPT isoform expression using bulk RNA sequencing data from xenografted human neurons. Data is represented as a 3R/4R ratio. Ratios greater than one represent higher 3R *MAPT* isoform expression, whereas ratio close to one represent a 50:50 expression of 3R and 4R *MAPT* isoforms. Ns, not significant.

Supplementary Figure 6:

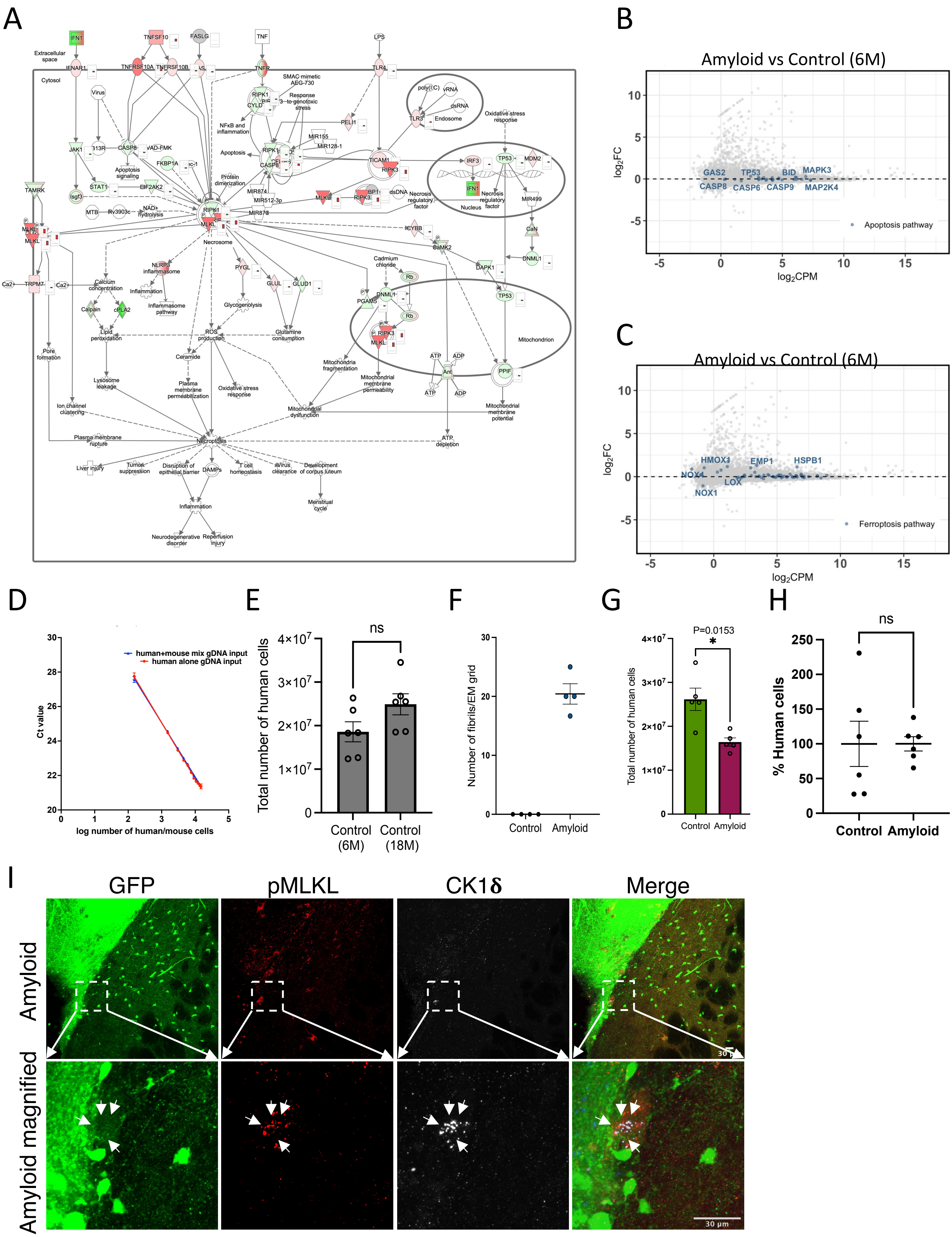

Supplementary Figure 6: Neuronal cell death analysis in transplanted human neurons.
(A) Ingenuity pathway analysis (IPA) of canonical necroptosis pathway overlaid with differentially expressed gene from the 6-months old neuronal xenografts. Red indicates upregulation, and green indicates downregulation. **(B)** Bland-Altman MA plot showing expression of apoptosis pathway genes in the 6 month old neuronal xenografts. **(C)** Bland-Altman MA plot showing expression of ferroptosis pathway genes in the 6 month old neuronal xenografts. **(D)** Establishment of the standard curve for qPCR using *TITIN* with human genomic DNA (red line) and human plus mouse genomic DNA (blue line). **(E)** Quantitative representation of the human neurons in control mice (6M and 18M) (n=6) using qPCR. **(F)** Quantification of the number of immunogold positive fibrils identified in the grafted control (n=4) and amyloid mice (n=4). **(G)** Quantitative representation of the human neurons at 18M post-transplantation in control (n=5) and amyloid (n=6) mice using qPCR. **(H)** Quantitative representation of the human neurons at 2M post-transplantation in control (n=6) and amyloid (n=6) mice using qPCR. **(I)**Representative confocal images showing co-localization of pMLKL with granulovacuolar degeneration (GVD) marker casein kinase 1 delta (CK1$\boldsymbol{\delta}$) in 18M old amyloid mice. Human graft (GFP in green), pMLKL (red), CK1$\boldsymbol{\delta}$ (grey). Scale bar 30 µm. Values are presented as mean ± SEM. Student's t-test used in E,G, and H to measure the statistical significance.

Supplementary Figure 7:

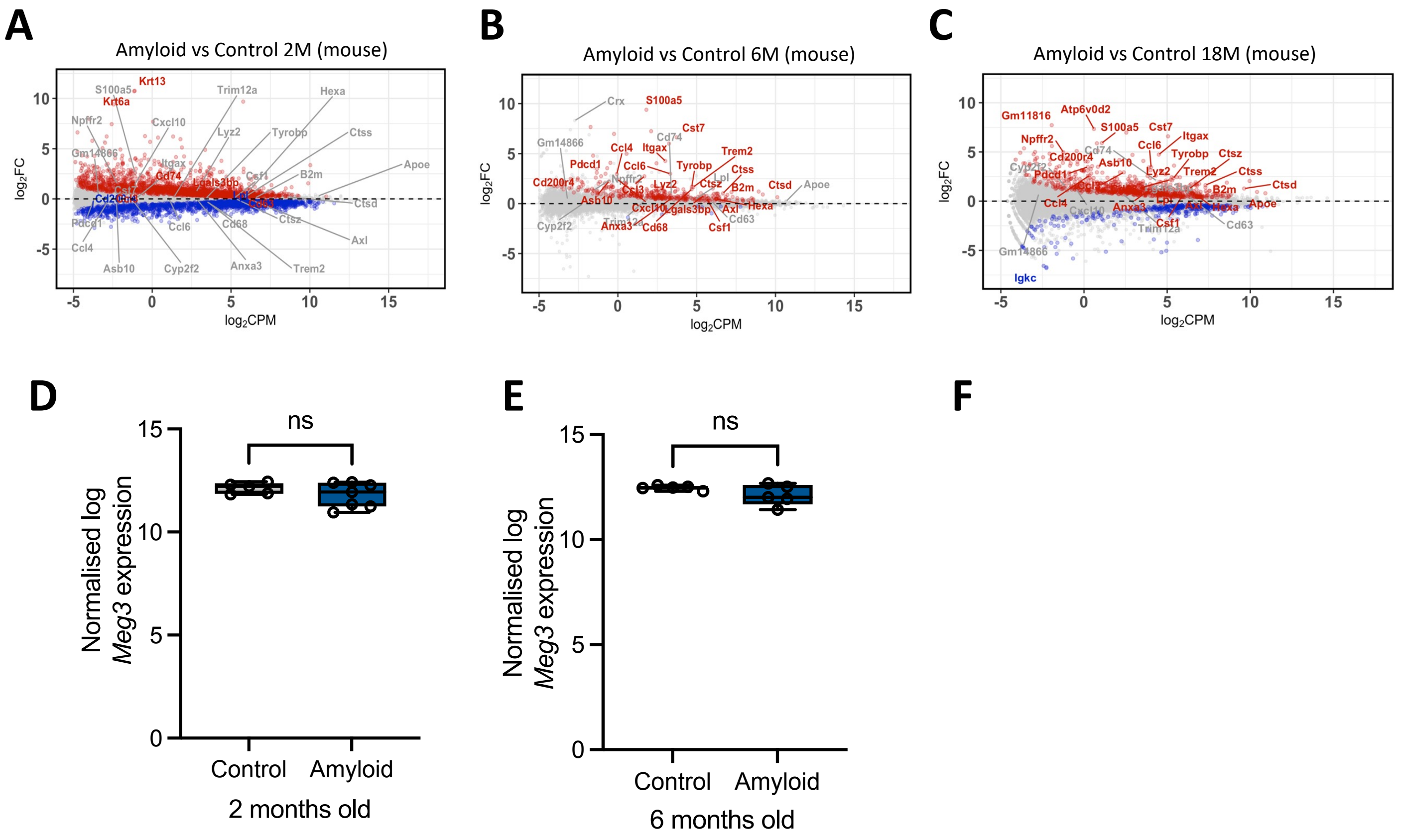

Supplementary Figure 7: Gene expression profiles of mouse host tissue from grafted mice at 2, 6 and 18 months.

(A) Bland-Altman MA plot showing the differential gene expression of mouse transcriptome at 2-months (control n=5, amyloid n=7), **(B),** 6-months (control n=5, amyloid n=5), and **(C),** 18-months (control n=4, amyloid n=3) old mice. Red, significantly upregulated genes. Blue, significantly downregulated genes (FDR < 0.05). Selected microglial activation genes are labelled. FC=fold change, CPM=counts per million. Analysis of mouse Meg3 expression 2- **(D)**, 6- **(E)** and 18 **(F)** months post transplantation. Bulk RNA sequencing reads from the RNA sequencing is extracted from the 2-, 6-, and 18- months old animals and presented as normalized counts (log scale) from either control or amyloid animals. ns, not significant. Values are presented as mean ± SEM. Student's t-test used in D, E and F to measure the statistical significance.

Supplementary Figure 8:

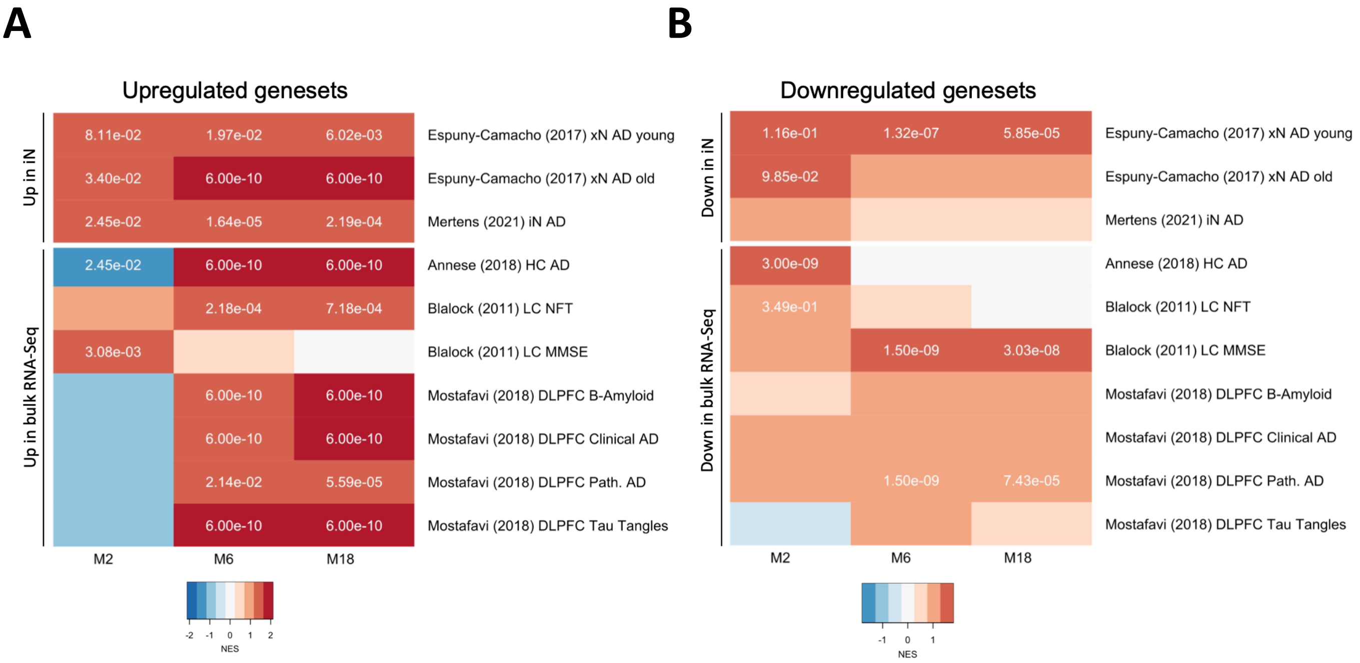

Supplementary Figure 8: Xenografted neurons recapitulate signatures identified in human post-mortem tissue and other xenografted neurons.

(A) Heatmap of normalized enrichment scores (NES) in indicated gene sets ranked along the human graft fold changes (upregulated genes, amyloid vs control) shown in figure 2a-c. Positive enrichments are shown in red, negative enrichments are shown in blue. Significant FDR values (p.adj < 0.01) are shown as text in the boxes. M2, upregulated genes in human neurons 2 months post-transplantation (PT). M6, upregulated genes in human neurons 6M PT. M18, upregulated genes in human neurons 18M PT. **(B)** Heatmap of normalised enrichment scores (NES) in indicated gene sets ranked along the human graft fold changes (downregulated genes, amyloid vs control) shown in figure 2a-c. Positive enrichments are shown in red, negative enrichments are shown in blue. Significant FDR values (p.adj < 0.05) are shown as text. M2, downregulated genes in human neurons 2 months post-transplantation (PT). M6, downregulated genes in human neurons 6M PT. M18, downregulated genes in human neurons 18M PT.

Datasets used in the above comparison:

Espuny-Camacho et al., 2017 xN AD young, DE upregulated genes form human neurons 4 months post-transplantation (PT). Espuny-Camacho et al., 2017 xN AD old, 6M-8M PT. (PMID: **28238547**). Martens et al., 2021 iN AD, DE genes from neurons directly reprogrammed from the fibroblast-derived from AD patients compared to neurons reprogrammed from control fibroblasts. (PMID: **33910058**). Annese (2018) HC AD, DE genes from Late-Onset AD (LOAD) compared to controls from the hippocampus (PMID: **29523845**). Blalock (2011) LC NFT, DE genes from laser captured P-Tau positive neurons. (PMID: **21756998**). Blalock (2004) MMSE, DE genes from MiniMental Status Examination (MMSE>20) from AD brains. (PMID: **14769913**). Mostafavi 2018 DLPFC, DE genes from dorsolateral prefrontal cortex from AD **(PMID: 29802388).**

Supplementary Figure 9:

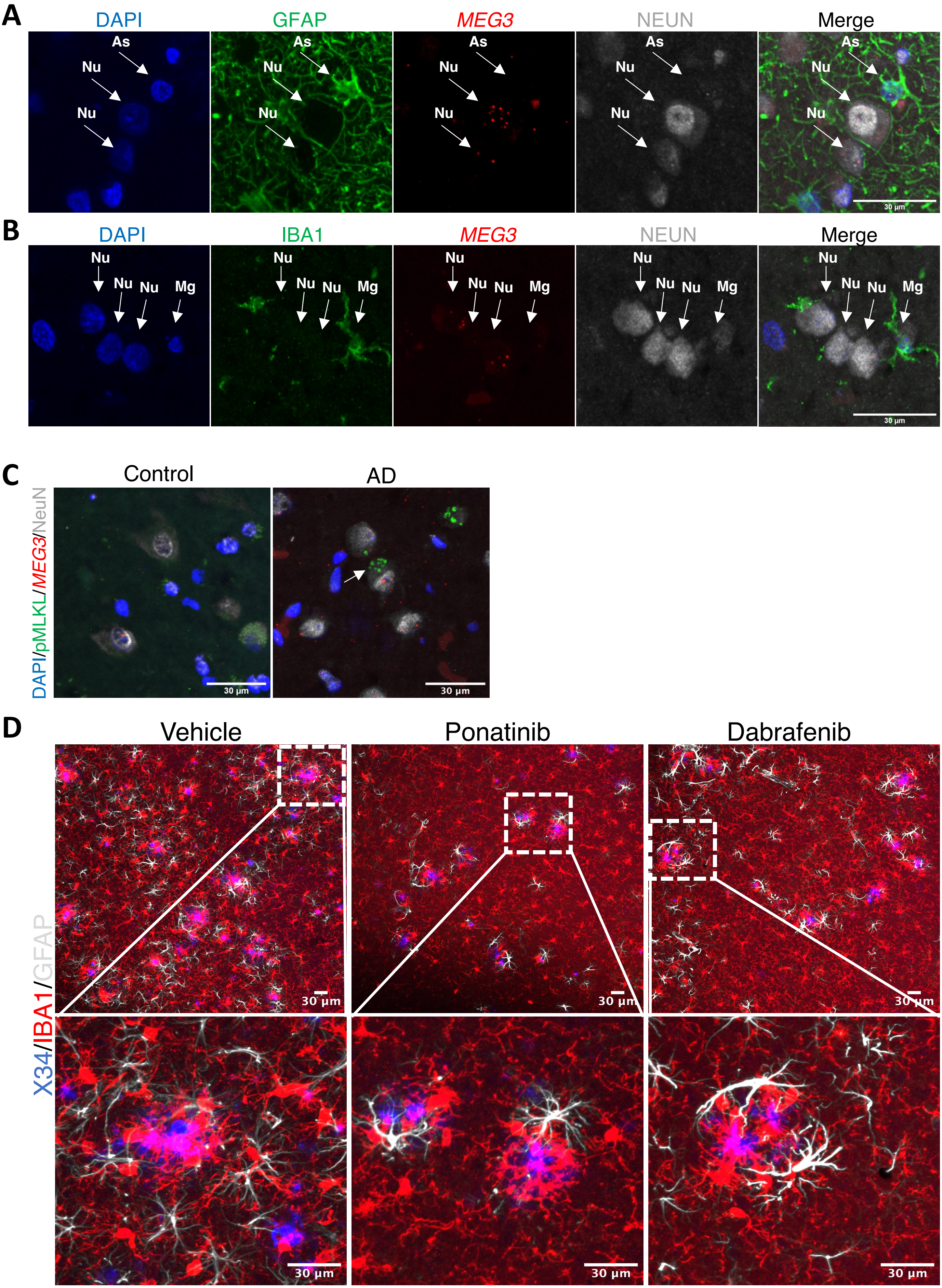

Supplementary Figure 9: *MEG3* is selectively expressed in neurons.

(A) Representative confocal images from FFPE fixed human hippocampal brain samples stained with *MEG3* (RNA scope, red) and combined with immunostaining for GFAP (green), NeuN (grey), and DAPI (blue). Nu=Neuron, As=Astrocytes. Scale bar 30 µm. **(B)** FFPE fixed human hippocampal brain samples were used for *MEG3* RNA scope (red) and combined with immunostaining for IBA1 (green), NeuN (grey), and DAPI (blue). Nu=Neuron, Mg=microglia. Scale bar 30 µm. **(C)** Representative confocal images showing the colocalization of pMLKL (green) in the *MEG3* positive neurons in AD (n=3) and control (n=3) hippocampal brain samples. Scale bar 30 µm. **(D)** Analysis of the glial cells from the mice administered with control diet (n=5) or diet mixed with Ponatinib (n=5) or Dabrafenib (n=6). Mice treated with the indicated drug from 2 months to 6 months added to the regular mouse maintenance diet. Brain samples were isolated after perfusion with PBS followed by 4% PFA post fixation overnight. Vibratome sections of 40 microns are made and stained with a X34 (blue, amyloid plaques), IBA1 (red, microglia), and GFAP (grey, astrocytes). Upper panel is a larger field of view from the cortex and the lower panel is a magnified field. Scale bar 30 µm.

Supplementary Figure 10:

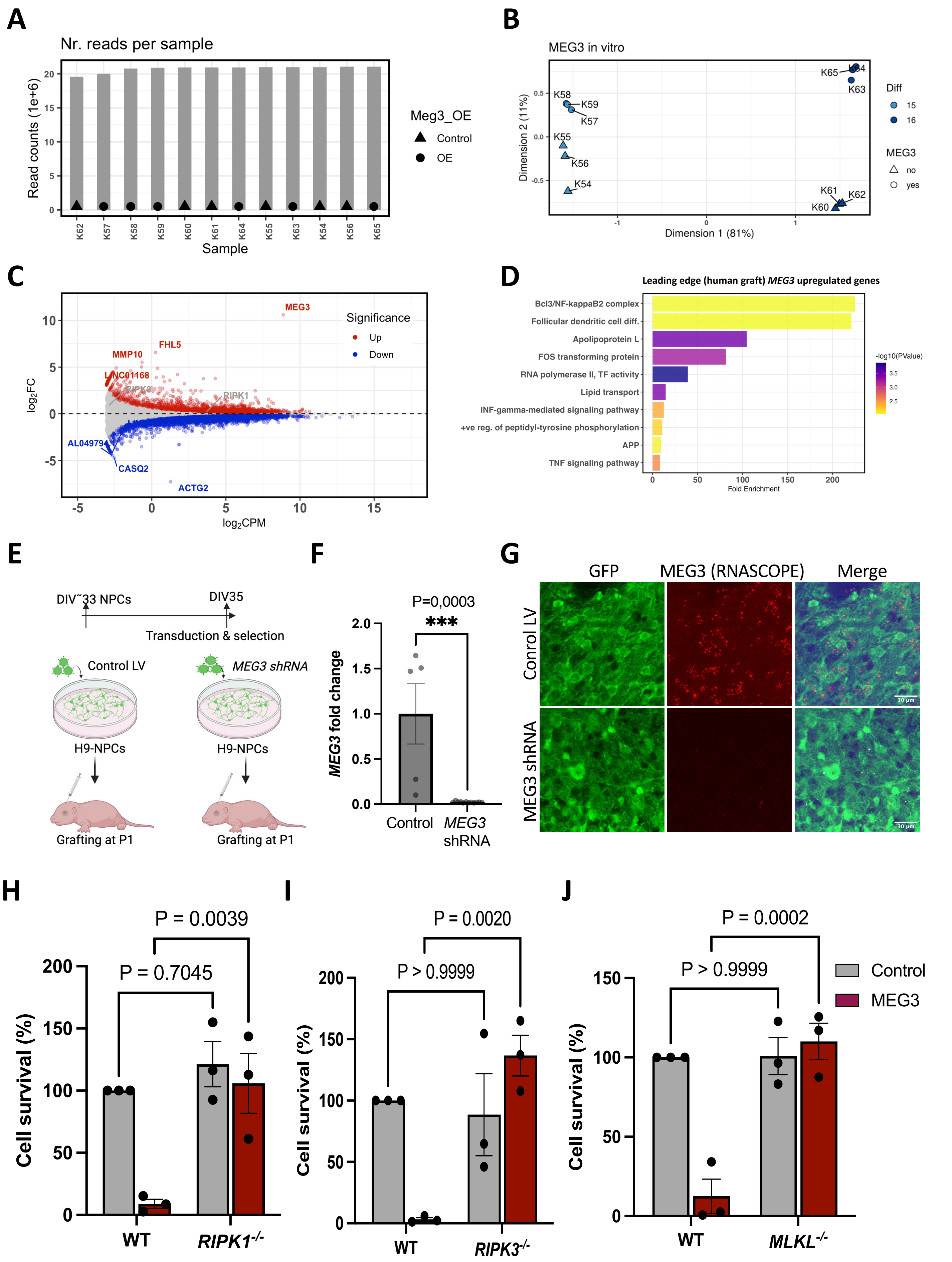

Supplementary Figure 10: RNA sequencing of the *MEG3* expressing neurons.

(A) Barplots of the total number of reads per sample obtained from RNA sequencing of *MEG3* oexpression (OE, control n=6, overexpression n=6). **(B)** Multi-dimensional scaling (MDS) of the RNA sequencing samples of *MEG3* expression (OE). Neurons derived from two independent differentiations (Diff), control LV (n=6), *MEG3* LV (n=6). **(C)** Bland-Altman MA plot showing differential expression of bulk RNA sequencing of *MEG3* expression (control LC n=6, *MEG3 LV* n=6). Significantly upregulated genes are shown in red and downregulated genes in blue (FDR < 0.05). Full differential expression results are shown in Supplementary Table 6. **(D)** DAVID gene ontology analysis of leading-edge upregulated genes from the GSEA analysis (indicated with red color in fig.3h). Top 10 GO terms were displayed. -ve=negative, +ve=positive, reg.=regulation , TF=transcription factor, TNF=tumor necrosis factor, APP=antigen process and presentation. **(E)** Schematic representation of the *MEG3* knockdown approach using *MEG3* shRNA. Lentiviral vectors containing the *MEG3* shRNA sequence were cloned into the lentiviral backbone and packaged. H9-derived NPCs were transduced with either the control GFP lentivirus or the *MEG3* shRNA, followed by puromycin selection to select positively transduced cells. Transduced cells were then transplanted into amyloid mice. **(F)** qRT-PCR analysis was conducted to assess human *MEG3* expression using RNA extracted from xenografted mouse brains at six months of age (control n=5; MEG3 shRNA n=13). **(G)** *MEG3* knockdown validation was performed using RNA Scope analysis on samples obtained from transplanted animals at six months of age. The white arrow indicates the *MEG3* RNA Scope puncta (red). Analysis of necroptosis inhibition in vitro using the RIPK1, RIPK3, and MLKL knock-out cells. Neuroprogenitor cells (NPCs), derived from the H9 stem cell line transduced with RIPK1 **(H)**, RIPK3 **(I)**, and MLKL **(J)**, were plated in flat-bottom black 96-well plates. Cells were treated with doxycycline to induce Cas9 in the indicated conditions. On approximately day 60 in vitro (~DIV60), the cells were transduced with either a control lentivirus or MEG3 lentivirus and cells were maintained in the neuronal maintenance media (NMM) until the CellTiter-Glo® assay. Ten days post transduction for the MEG3 CellTiter-Glo® luminescent cell viability assay was performed to measure cell viability. The assay was conducted in triplicates with six technical replicates for each replicate. The data from the control wells were normalized, and the results are presented as the percentage of cell survival between the control and treated conditions. Values are presented as mean ± SEM. Two-way ANOVA with Bonferroni multiple comparison test (H, I and J) and t-test (F) was used for measuring the statistical significance.

Supplementary tables

Supplementary Table -1: This table contains data on the differential gene expression obtained from the bulk RNA transcriptome analysis of human grafts from 2-, 6-, and 18-month-old control and amyloid. Animals. The data presented here correspond to the main figures 2A-D.

Supplementary Table -2: This table contains the results of the differential gene expression analysis data from the bulk RNA transcriptome of the mouse host tissue from 2-, 6-, and 18-month-old control and amyloid grafted. The data presented here correspond to supplementary figures 7A-C.

Supplementary Table -3: This table comprises datasets previously published in the literature, which were utilized in supplementary figure 8 and the main figure 2E.

Supplementary Table -4: The data in this table related to the differential gene expression gene data obtained from the bulk RNA transcriptome analysis of neurons transduced with either control lentivirus or MEG3 lentiviral vector. The data presented here correspond to supplementary figures 10C-D.

Supplementary Table -5: This table provides details about the key resources, materials, and antibodies used in the study, along with their respective identifiers.

References and Notes

1. Z. He, J. L. Guo, J. D. McBride, S. Narasimhan, H. Kim, L. Changolkar, B. Zhang, R. J. Gathagan, C. Yue, C. Dengler, A. Stieber, M. Nitla, D. A. Coulter, T. Abel, K. R. Brunden, J. Q. Trojanowski, V. M. Y. Lee, Amyloid-β plaques enhance Alzheimer's brain tau-seeded pathologies by facilitating neuritic plaque tau aggregation. *Nat. Med.* **24**, 29–38 (2018). doi:10.1038/nm.4443 Medline

2. F. Grueninger, B. Bohrmann, C. Czech, T. M. Ballard, J. R. Frey, C. Weidensteiner, M. von Kienlin, L. Ozmen, Phosphorylation of Tau at S422 is enhanced by Aβ in TauPS2APP triple transgenic mice. *Neurobiol. Dis.* **37**, 294–306 (2010). doi:10.1016/j.nbd.2009.09.004 Medline

3. N. Mattsson-Carlgren, E. Andersson, S. Janelidze, R. Ossenkoppele, P. Insel, O. Strandberg, H. Zetterberg, H. J. Rosen, G. Rabinovici, X. Chai, K. Blennow, J. L. Dage, E. Stomrud, R. Smith, S. Palmqvist, O. Hansson, Aβ deposition is associated with increases in soluble and phosphorylated tau that precede a positive Tau PET in Alzheimer's disease. *Sci. Adv.* **6**, eaaz2387 (2020). doi:10.1126/sciadv.aaz2387 Medline

4. C. Guela, C.-K. Wu, D. Saroff, A. Lorenzo, M. Yuan, B. A. Yankner, Aging renders the brain vulnerable to amyloid β-protein neurotoxicity. *Nat. Med.* **4**, 827–831 (1998). doi:10.1038/nm0798-827 Medline

5. S. H. Choi, Y. H. Kim, M. Hebisch, C. Sliwinski, S. Lee, C. D'Avanzo, H. Chen, B. Hooli, C. Asselin, J. Muffat, J. B. Klee, C. Zhang, B. J. Wainger, M. Peitz, D. M. Kovacs, C. J. Woolf, S. L. Wagner, R. E. Tanzi, D. Y. Kim, A three-dimensional human neural cell culture model of Alzheimer's disease. *Nature* **515**, 274–278 (2014). doi:10.1038/nature13800 Medline

6. I. Espuny-Camacho, A. M. Arranz, M. Fiers, A. Snellinx, K. Ando, S. Munck, J. Bonnefont, L. Lambot, N. Corthout, L. Omodho, E. Vanden Eynden, E. Radaelli, I. Tesseur, S. Wray, A. Ebneth, J. Hardy, K. Leroy, J. P. Brion, P. Vanderhaeghen, B. De Strooper, Hallmarks of Alzheimer's disease in stem-cell-derived human neurons transplanted into mouse brain. *Neuron* **93**, 1066–1081.e8 (2017). doi:10.1016/j.neuron.2017.02.001 Medline

7. S. Janelidze, N. Mattsson, S. Palmqvist, R. Smith, T. G. Beach, G. E. Serrano, X. Chai, N. K. Proctor, U. Eichenlaub, H. Zetterberg, K. Blennow, E. M. Reiman, E. Stomrud, J. L. Dage, O. Hansson, Plasma P-tau181 in Alzheimer's disease: relationship to other biomarkers, differential diagnosis, neuropathology and longitudinal progression to Alzheimer's dementia. *Nat. Med.* **26**, 379–386 (2020). doi:10.1038/s41591-020-0755-1 Medline

8. E. H. Thijssen, R. La Joie, A. Wolf, A. Strom, P. Wang, L. Iaccarino, V. Bourakova, Y. Cobigo, H. Heuer, S. Spina, L. VandeVrede, X. Chai, N. K. Proctor, D. C. Airey, S. Shcherbinin, C. Duggan Evans, J. R. Sims, H. Zetterberg, K. Blennow, A. M. Karydas, C. E. Teunissen, J. H. Kramer, L. T. Grinberg, W. W. Seeley, H. Rosen, B. F. Boeve, B. L. Miller, G. D. Rabinovici, J. L. Dage, J. C. Rojas, A. L. Boxer; Advancing Research and Treatment for Frontotemporal Lobar Degeneration (ARTFL) investigators, Diagnostic value of plasma phosphorylated tau181 in Alzheimer's disease and frontotemporal lobar degeneration. *Nat. Med.* **26**, 387–397 (2020). doi:10.1038/s41591-020-0762-2 Medline

9. M. Bensalah, P. Klein, I. Riederer, S. Chaouch, L. Muraine, W. Savino, G. S. Butler-Browne, C. Trollet, V. Mouly, A. Bigot, E. Negroni, Combined methods to evaluate human cells in muscle xenografts. *PLOS ONE* **14**, e0211522 (2019). doi:10.1371/journal.pone.0211522 Medline

10. S. E. Marsh, E. M. Abud, A. Lakatos, A. Karimzadeh, S. T. Yeung, H. Davtyan, G. M. Fote, L. Lau, J. G. Weinger, T. E. Lane, M. A. Inlay, W. W. Poon, M. Blurton-Jones, The adaptive immune system restrains Alzheimer's disease pathogenesis by modulating microglial function. *Proc. Natl. Acad. Sci. U.S.A.* **113**, E1316–E1325 (2016). doi:10.1073/pnas.1525466113 Medline

11. C. Späni, T. Suter, R. Derungs, M. T. Ferretti, T. Welt, F. Wirth, C. Gericke, R. M. Nitsch, L. Kulic, Reduced β-amyloid pathology in an APP transgenic mouse model of Alzheimer's disease lacking functional B and T cells. *Acta Neuropathol. Commun.* **3**, 71 (2015). doi:10.1186/s40478-015-0251-x Medline

12. A. Sierksma, A. Lu, R. Mancuso, N. Fattorelli, N. Thrupp, E. Salta, J. Zoco, D. Blum, L. Buée, B. De Strooper, M. Fiers, Novel Alzheimer risk genes determine the microglia response to amyloid-β but not to TAU pathology. *EMBO Mol. Med.* **12**, e10606 (2020). doi:10.15252/emmm.201910606 Medline

13. C. Sala Frigerio, L. Wolfs, N. Fattorelli, N. Thrupp, I. Voytyuk, I. Schmidt, R. Mancuso, W. T. Chen, M. E. Woodbury, G. Srivastava, T. Möller, E. Hudry, S. Das, T. Saido, E. Karran, B. Hyman, V. H. Perry, M. Fiers, B. De Strooper, The major risk factors for Alzheimer's disease: age, sex, and genes modulate the microglia response to Aβ plaques. *Cell Rep.* **27**, 1293–1306.e6 (2019). doi:10.1016/j.celrep.2019.03.099 Medline

14. K. J. Bryan, X. Zhu, P. L. Harris, G. Perry, R. J. Castellani, M. A. Smith, G. Casadesus, Expression of CD74 is increased in neurofibrillary tangles in Alzheimer's disease. *Mol. Neurodegener.* **3**, 13 (2008). doi:10.1186/1750-1326-3-13 Medline

15. M. Biagioli, M. Pinto, D. Cesselli, M. Zaninello, D. Lazarevic, P. Roncaglia, R. Simone, C. Vlachouli, C. Plessy, N. Bertin, A. Beltrami, K. Kobayashi, V. Gallo, C. Santoro, I. Ferrer, S. Rivella, C. A. Beltrami, P. Carninci, E. Raviola, S. Gustincich, Unexpected expression of α- and β-globin in mesencephalic dopaminergic neurons and glial cells. *Proc. Natl. Acad. Sci. U.S.A.* **106**, 15454–15459 (2009). doi:10.1073/pnas.0813216106 Medline

16. J. Y. Chuang, C. W. Lee, Y. H. Shih, T. Yang, L. Yu, Y. M. Kuo, Interactions between amyloid-β and hemoglobin: Implications for amyloid plaque formation in Alzheimer's disease. *PLOS ONE* **7**, e33120 (2012). doi:10.1371/journal.pone.0033120 Medline

17. N. Brown, K. Alkhayer, R. Clements, N. Singhal, R. Gregory, S. Azzam, S. Li, E. Freeman, J. McDonough, Neuronal hemoglobin expression and its relevance to multiple sclerosis neuropathology. *J. Mol. Neurosci.* **59**, 1–17 (2016). doi:10.1007/s12031-015-0711-6 Medline

18. D. Van Gool, B. De Strooper, F. Van Leuven, E. Triau, R. Dom, alpha 2-macroglobulin expression in neuritic-type plaques in patients with Alzheimer's disease. *Neurobiol. Aging* **14**, 233–237 (1993). doi:10.1016/0197-4580(93)90006-W Medline

19. W.-T. Chen, A. Lu, K. Craessaerts, B. Pavie, C. Sala Frigerio, N. Corthout, X. Qian, J. Laláková, M. Kühnemund, I. Voytyuk, L. Wolfs, R. Mancuso, E. Salta, S. Balusu, A. Snellinx, S. Munck, A. Jurek, J. Fernandez Navarro, T. C. Saido, I. Huitinga, J. Lundeberg, M. Fiers, B. De Strooper, Spatial transcriptomics and *in situ* sequencing to study Alzheimer's disease. *Cell* **182**, 976–991.e19 (2020). doi:10.1016/j.cell.2020.06.038 Medline

20. S. Mostafavi, C. Gaiteri, S. E. Sullivan, C. C. White, S. Tasaki, J. Xu, M. Taga, H. U. Klein, E. Patrick, V. Komashko, C. McCabe, R. Smith, E. M. Bradshaw, D. E. Root, A. Regev, L. Yu, L. B. Chibnik, J. A. Schneider, T. L. Young-Pearse, D. A. Bennett, P. L. De Jager, A molecular network of the aging human brain provides insights into the pathology and cognitive decline of Alzheimer's disease. *Nat. Neurosci.* **21**, 811–819 (2018). doi:10.1038/s41593-018-0154-9 Medline

21. J. Mertens, J. R. Herdy, L. Traxler, S. T. Schafer, J. C. M. Schlachetzki, L. Böhnke, D. A. Reid, H. Lee, D. Zangwill, D. P. Fernandes, R. K. Agarwal, R. Lucciola, L. Zhou-Yang, L. Karbacher, F. Edenhofer, S. Stern, S. Horvath, A. C. M. Paquola, C. K. Glass, S. H. Yuan, M. Ku, A. Szücs, L. S. B. Goldstein, D. Galasko, F. H. Gage, Age-dependent instability of mature neuronal fate in induced neurons from Alzheimer's patients. *Cell Stem Cell* **28**, 1533–1548.e6 (2021). doi:10.1016/j.stem.2021.04.004 Medline

22. T. Arendt, Cell cycle activation and aneuploid neurons in Alzheimer's disease. *Mol. Neurobiol.* **46**, 125–135 (2012). doi:10.1007/s12035-012-8262-0 Medline

23. T. Arendt, M. Holzer, A. Stöbe, U. Gärtner, H. J. Lüth, M. K. Brückner, U. Ueberham, Activated mitogenic signaling induces a process of dedifferentiation in Alzheimer's disease that eventually results in cell death. *Ann. N. Y. Acad. Sci.* **920**, 249–255 (2000). doi:10.1111/j.1749-6632.2000.tb06931.x Medline

24. Y. Yang, D. S. Geldmacher, K. Herrup, DNA replication precedes neuronal cell death in Alzheimer's disease. *J. Neurosci.* **21**, 2661–2668 (2001). doi:10.1523/JNEUROSCI.21-08-02661.2001 Medline

25. O. Wirths, S. Zampar, Neuron loss in Alzheimer's disease: Translation in transgenic mouse models. *Int. J. Mol. Sci.* **21**, 8144 (2020). doi:10.3390/ijms21218144 Medline

26. U. Fünfschilling, L. M. Supplie, D. Mahad, S. Boretius, A. S. Saab, J. Edgar, B. G. Brinkmann, C. M. Kassmann, I. D. Tzvetanova, W. Möbius, F. Diaz, D. Meijer, U. Suter, B. Hamprecht, M. W. Sereda, C. T. Moraes, J. Frahm, S. Goebbels, K. A. Nave, Glycolytic oligodendrocytes maintain myelin and long-term axonal integrity. *Nature* **485**, 517–521 (2012). doi:10.1038/nature11007 Medline

27. M. J. Koper, E. Van Schoor, S. Ospitalieri, R. Vandenberghe, M. Vandenbulcke, C. A. F. von Arnim, T. Tousseyn, S. Balusu, B. De Strooper, D. R. Thal, Necrosome complex detected in granulovacuolar degeneration is associated with neuronal loss in Alzheimer's disease. *Acta Neuropathol.* **139**, 463–484 (2020). doi:10.1007/s00401-019-02103-y Medline

28. E. Salta, B. De Strooper, Non-coding RNAs with essential roles in neurodegenerative disorders. *Lancet Neurol.* **11**, 189–200 (2012). doi:10.1016/S1474-4422(11)70286-1 Medline

29. N. Jiang, X. Zhang, X. Gu, X. Li, L. Shang, Progress in understanding the role of lncRNA in programmed cell death. *Cell Death Discov.* **7**, 30 (2021). doi:10.1038/s41420-021-00407-1 Medline

30. Y. Zhou, Y. Zhong, Y. Wang, X. Zhang, D. L. Batista, R. Gejman, P. J. Ansell, J. Zhao, C. Weng, A. Klibanski, Activation of p53 by MEG3 non-coding RNA. *J. Biol. Chem.* **282**, 24731–24742 (2007). doi:10.1074/jbc.M702029200 Medline

31. T. Mondal, S. Subhash, R. Vaid, S. Enroth, S. Uday, B. Reinius, S. Mitra, A. Mohammed, A. R. James, E. Hoberg, A. Moustakas, U. Gyllensten, S. J. M. Jones, C. M. Gustafsson, A. H. Sims, F. Westerlund, E. Gorab, C. Kanduri, MEG3 long noncoding RNA regulates the TGF-β pathway genes through formation of RNA-DNA triplex structures. *Nat. Commun.* **6**, 7743 (2015). doi:10.1038/ncomms8743 Medline

32. K. Chanda, S. Das, J. Chakraborty, S. Bucha, A. Maitra, R. Chatterjee, D. Mukhopadhyay, N. P. Bhattacharyya, Altered levels of long ncRNAs Meg3 and Neat1 in cell and animal models of Huntington's disease. *RNA Biol.* **15**, 1348–1363 (2018). doi:10.1080/15476286.2018.1534524 Medline

33. A. Grubman, G. Chew, J. F. Ouyang, G. Sun, X. Y. Choo, C. McLean, R. K. Simmons, S. Buckberry, D. B. Vargas-Landin, D. Poppe, J. Pflueger, R. Lister, O. J. L. Rackham, E. Petretto, J. M. Polo, A single-cell atlas of entorhinal cortex from individuals with Alzheimer's disease reveals cell-type-specific gene expression regulation. *Nat. Neurosci.* **22**, 2087–2097 (2019). doi:10.1038/s41593-019-0539-4 Medline

34. Y. Zhou, X. Zhang, A. Klibanski, MEG3 noncoding RNA: A tumor suppressor. *J. Mol. Endocrinol.* **48**, R45–R53 (2012). doi:10.1530/JME-12-0008 Medline

35. A. Jayaraman, T. T. Htike, R. James, C. Picon, R. Reynolds, TNF-mediated neuroinflammation is linked to neuronal necroptosis in Alzheimer's disease hippocampus. *Acta Neuropathol. Commun.* **9**, 159 (2021). doi:10.1186/s40478-021-01264-w Medline

36. H. Zhang, Mechanism associated with aberrant lncRNA MEG3 expression in gestational diabetes mellitus. *Exp. Ther. Med.* **18**, 3699–3706 (2019). doi:10.3892/etm.2019.8062 Medline

37. H. Bi, G. Wang, Z. Li, L. Zhou, M. Zhang, J. Ye, Z. Wang, Long noncoding RNA (lncRNA) maternally expressed gene 3 (MEG3) participates in chronic obstructive pulmonary disease through regulating human pulmonary microvascular endothelial cell apoptosis. *Med. Sci. Monit.* **26**, e920793 (2020). doi:10.12659/MSM.920793 Medline

38. A. Fauster, M. Rebsamen, K. V. M. Huber, J. W. Bigenzahn, A. Stukalov, C. H. Lardeau, S. Scorzoni, M. Bruckner, M. Gridling, K. Parapatics, J. Colinge, K. L. Bennett, S. Kubicek, S. Krautwald, A. Linkermann, G. Superti-Furga, A cellular screen identifies ponatinib and pazopanib as inhibitors of necroptosis. *Cell Death Dis.* **6**, e1767 (2015). doi:10.1038/cddis.2015.130 Medline

39. J.-X. Li, J.-M. Feng, Y. Wang, X.-H. Li, X.-X. Chen, Y. Su, Y.-Y. Shen, Y. Chen, B. Xiong, C.-H. Yang, J. Ding, Z.-H. Miao, The B-Raf^{V600E} inhibitor dabrafenib selectively inhibits RIP3 and alleviates acetaminophen-induced liver injury. *Cell Death Dis.* **5**, e1278 (2014). doi:10.1038/cddis.2014.241 Medline

40. S. Martens, S. Hofmans, W. Declercq, K. Augustyns, P. Vandenabeele, Inhibitors targeting RIPK1/RIPK3: old and new drugs. *Trends Pharmacol. Sci.* **41**, 209–224 (2020). doi:10.1016/j.tips.2020.01.002 Medline

41. K. Newton, K. E. Wickliffe, A. Maltzman, D. L. Dugger, A. Strasser, V. C. Pham, J. R. Lill, M. Roose-Girma, S. Warming, M. Solon, H. Ngu, J. D. Webster, V. M. Dixit, RIPK1 inhibits ZBP1-driven necroptosis during development. *Nature* **540**, 129–133 (2016). doi:10.1038/nature20559 Medline

42. K. A. Roth, Caspases, apoptosis, and Alzheimer disease: Causation, correlation, and confusion. *J. Neuropathol. Exp. Neurol.* **60**, 829–838 (2001). doi:10.1093/jnen/60.9.829 Medline

43. A. Caccamo, C. Branca, I. S. Piras, E. Ferreira, M. J. Huentelman, W. S. Liang, B. Readhead, J. T. Dudley, E. E. Spangenberg, K. N. Green, R. Belfiore, W. Winslow, S. Oddo, Necroptosis activation in Alzheimer's disease. *Nat. Neurosci.* **20**, 1236–1246 (2017). doi:10.1038/nn.4608 Medline

44. B. De Strooper, E. Karran, The cellular phase of Alzheimer's disease. *Cell* **164**, 603–615 (2016). doi:10.1016/j.cell.2015.12.056 Medline

45. A. Sierksma, V. Escott-Price, B. De Strooper, Translating genetic risk of Alzheimer's disease into mechanistic insight and drug targets. *Science* **370**, 61–66 (2020). doi:10.1126/science.abb8575 Medline

46. E. E. Congdon, E. M. Sigurdsson, Tau-targeting therapies for Alzheimer disease. *Nat. Rev. Neurol.* **14**, 399–415 (2018). doi:10.1038/s41582-018-0013-z Medline

47. J. Sevigny, P. Chiao, T. Bussière, P. H. Weinreb, L. Williams, M. Maier, R. Dunstan, S. Salloway, T. Chen, Y. Ling, J. O'Gorman, F. Qian, M. Arastu, M. Li, S. Chollate, M. S. Brennan, O. Quintero-Monzon, R. H. Scannevin, H. M. Arnold, T. Engber, K. Rhodes, J. Ferrero, Y. Hang, A. Mikulskis, J. Grimm, C. Hock, R. M. Nitsch, A. Sandrock, The antibody aducanumab reduces Aβ plaques in Alzheimer's disease. *Nature* **537**, 50–56 (2016). doi:10.1038/nature19323 Medline

48. M. A. Mintun, A. C. Lo, C. Duggan Evans, A. M. Wessels, P. A. Ardayfio, S. W. Andersen, S. Shcherbinin, J. Sparks, J. R. Sims, M. Brys, L. G. Apostolova, S. P. Salloway, D. M. Skovronsky, Donanemab in early Alzheimer's disease. *N. Engl. J. Med.* **384**, 1691–1704 (2021). doi:10.1056/NEJMoa2100708 Medline

49. Y. Ito, D. Ofengeim, A. Najafov, S. Das, S. Saberi, Y. Li, J. Hitomi, H. Zhu, H. Chen, L. Mayo, J. Geng, P. Amin, J. P. DeWitt, A. K. Mookhtiar, M. Florez, A. T. Ouchida, J. B. Fan, M. Pasparakis, M. A. Kelliher, J. Ravits, J. Yuan, RIPK1 mediates axonal degeneration by promoting inflammation and necroptosis in ALS. *Science* **353**, 603–608 (2016). doi:10.1126/science.aaf6803 Medline

50. J. Yuan, P. Amin, D. Ofengeim, Necroptosis and RIPK1-mediated neuroinflammation in CNS diseases. *Nat. Rev. Neurosci.* **20**, 19–33 (2019). doi:10.1038/s41583-018-0093-1 Medline

51. B. Hanson, Necroptosis: A new way of dying? *Cancer Biol. Ther.* **17**, 899–910 (2016). doi:10.1080/15384047.2016.1210732 Medline

52. P. J. Rugg-Gunn, A. C. Ferguson-Smith, R. A. Pedersen, Status of genomic imprinting in human embryonic stem cells as revealed by a large cohort of independently derived and maintained lines. *Hum. Mol. Genet.* **16**, R243–R251 (2007). doi:10.1093/hmg/ddm245 Medline

53. E. Lauretti, K. Dabrowski, D. Praticò, The neurobiology of non-coding RNAs and Alzheimer's disease pathogenesis: Pathways, mechanisms and translational opportunities. *Ageing Res. Rev.* **71**, 101425 (2021). doi:10.1016/j.arr.2021.101425 Medline

54. F. Supek, M. Bošnjak, N. Škunca, T. Šmuc, REVIGO summarizes and visualizes long lists of gene ontology terms. *PLOS ONE* **6**, e21800 (2011). doi:10.1371/journal.pone.0021800 Medline

55. Y. Shi, P. Kirwan, F. J. Livesey, Directed differentiation of human pluripotent stem cells to cerebral cortex neurons and neural networks. *Nat. Protoc.* **7**, 1836–1846 (2012). doi:10.1038/nprot.2012.116 Medline

56. N. Gaspard, T. Bouschet, A. Herpoel, G. Naeije, J. van den Ameele, P. Vanderhaeghen, Generation of cortical neurons from mouse embryonic stem cells. *Nat. Protoc.* **4**, 1454–1463 (2009). doi:10.1038/nprot.2009.157 Medline

57. N. Kuninaka, M. Kawaguchi, M. Ogawa, A. Sato, K. Arima, S. Murayama, Y. Saito, Simplification of the modified Gallyas method. *Neuropathology* **35**, 10–15 (2015). doi:10.1111/neup.12144 Medline

58. J. L. Guo, S. Narasimhan, L. Changolkar, Z. He, A. Stieber, B. Zhang, R. J. Gathagan, M. Iba, J. D. McBride, J. Q. Trojanowski, V. M. Y. Lee, Unique pathological tau conformers from Alzheimer's brains transmit tau pathology in nontransgenic mice. *J. Exp. Med.* **213**, 2635–2654 (2016). doi:10.1084/jem.20160833 Medline

59. B. Falcon, W. Zhang, M. Schweighauser, A. G. Murzin, R. Vidal, H. J. Garringer, B. Ghetti, S. H. W. Scheres, M. Goedert, Tau filaments from multiple cases of sporadic and inherited Alzheimer's disease adopt a common fold. *Acta Neuropathol.* **136**, 699–708 (2018). doi:10.1007/s00401-018-1914-z Medline

60. S. Y. Alcoser, D. J. Kimmel, S. D. Borgel, J. P. Carter, K. M. Dougherty, M. G. Hollingshead, Real-time PCR-based assay to quantify the relative amount of human and mouse tissue present in tumor xenografts. *BMC Biotechnol.* **11**, 124 (2011). doi:10.1186/1472-6750-11-124 Medline

61. P. Song, Z. Xie, L. Guo, C. Wang, W. Xie, Y. Wu, Human genome-specific real-time PCR method for sensitive detection and reproducible quantitation of human cells in mice. *Stem Cell Rev. Rep.* **8**, 1155–1162 (2012). doi:10.1007/s12015-012-9406-3 Medline

62. A. Dobin, C. A. Davis, F. Schlesinger, J. Drenkow, C. Zaleski, S. Jha, P. Batut, M. Chaisson, T. R. Gingeras, STAR: Ultrafast universal RNA-seq aligner. *Bioinformatics* **29**, 15–21 (2013). doi:10.1093/bioinformatics/bts635 Medline

63. Y. Liao, G. K. Smyth, W. Shi, featureCounts: An efficient general purpose program for assigning sequence reads to genomic features. *Bioinformatics* **30**, 923–930 (2014). doi:10.1093/bioinformatics/btt656 Medline

64. M. D. Robinson, D. J. McCarthy, G. K. Smyth, edgeR: A Bioconductor package for differential expression analysis of digital gene expression data. *Bioinformatics* **26**, 139–140 (2010). doi:10.1093/bioinformatics/btp616 Medline

65. M. E. Ritchie, B. Phipson, D. Wu, Y. Hu, C. W. Law, W. Shi, G. K. Smyth, *limma* powers differential expression analyses for RNA-sequencing and microarray studies. *Nucleic Acids Res.* **43**, e47 (2015). doi:10.1093/nar/gkv007 Medline

66. W. Huang, B. T. Sherman, R. A. Lempicki, Systematic and integrative analysis of large gene lists using DAVID bioinformatics resources. *Nat. Protoc.* **4**, 44–57 (2009). doi:10.1038/nprot.2008.211 Medline

67. W. Huang, B. T. Sherman, R. A. Lempicki, Bioinformatics enrichment tools: Paths toward the comprehensive functional analysis of large gene lists. *Nucleic Acids Res.* **37**, 1–13 (2009). doi:10.1093/nar/gkn923 Medline

68. I. Espuny-Camacho, K. A. Michelsen, D. Gall, D. Linaro, A. Hasche, J. Bonnefont, C. Bali, D. Orduz, A. Bilheu, A. Herpoel, N. Lambert, N. Gaspard, S. Péron, S. N. Schiffmann, M. Giugliano, A. Gaillard, P. Vanderhaeghen, Pyramidal neurons derived from human pluripotent stem cells integrate efficiently into mouse brain circuits in vivo. *Neuron* **77**, 440–456 (2013). doi:10.1016/j.neuron.2012.12.011 Medline

69. X. Zheng, L. Boyer, M. Jin, J. Mertens, Y. Kim, L. Ma, L. Ma, M. Hamm, F. H. Gage, T. Hunter, Metabolic reprogramming during neuronal differentiation from aerobic glycolysis to neuronal oxidative phosphorylation. *eLife* **5**, e13374 (2016). doi:10.7554/eLife.13374 Medline
